# Nanomaterial modulation of lysosomal homeostasis in neurodegenerative proteinopathies: from pathological-protein degradation to re-release and seeding

**DOI:** 10.3389/fnmol.2026.1997252

**Published:** 2026-10-19

**Authors:** Mingrui Song, Nan Bu, Long Wei, Jing Chen

**Affiliations:** 1 The Second Affiliated Hospital of Heilongjiang University of Chinese Medicine, Harbin, China; 2 Heilongjiang University of Chinese Medicine, Harbin, China

**Keywords:** autophagy, extracellular vesicles, lysosomal acidification, lysosomal homeostasis, nanomaterials, neurodegenerative proteinopathies, protein seeding

## Abstract

Misfolded and aggregated proteins are central to neurodegenerative proteinopathies, yet a reduction in intracellular protein burden does not necessarily indicate productive clearance, because incompletely processed cargo may be re-released and retain seeding competence. This review examines how nanomaterials targeting lysosomal homeostasis may promote complete degradation of pathological proteins while limiting the re-release of incompletely processed species and their residual seeding activity. PubMed records published from July 2021 to July 2026 were prioritised, with earlier foundational studies included selectively, and evidence was classified as direct disease-model evidence, mechanistic support, or platform extrapolation. Across Aβ, tau, α-synuclein, polyglutamine-expanded proteins, and SOD1-related pathology, effective clearance depends on protein conformation, cell type, lysosomal acidification and hydrolase activity, autophagic transport, lipid processing, membrane integrity, and cellular bioenergetics. Current evidence is strongest for restoration of lysosomal acidity, hydrolase function, and autophagic processing; support for durable lipid and membrane correction is less complete, and direct evidence for reduced re-release and seed inactivation remains scarce. No reviewed study simultaneously demonstrated lysosomal restoration, complete degradation of a defined pathological substrate, reduced extracellular release, and direct seed inactivation. Future evaluation should therefore integrate brain, cell-specific and organelle delivery, substrate turnover, extracellular cargo, direct seeding assays, and repeated-dose lysosomal safety.

## 1 Introduction

The accumulation of misfolded and aggregated proteins is a shared feature of many neurodegenerative disorders, but the proteins involved do not constitute a single, uniform therapeutic target. Amyloid-β (Aβ), tau, α-synuclein, polyglutamine-expanded proteins and superoxide dismutase 1 (SOD1) differ in their predominant cellular location, post-translational state, membrane association and tendency to form soluble oligomers, fibrils or larger inclusions. These distinctions are more than descriptive. They influence how pathological proteins are recognised, internalised, transported and processed by neural cells. Misfolded proteins may exert local toxicity by disrupting membranes, organelle trafficking and proteostasis, while certain conformational states can also act as seeds that induce further aggregation after transfer to neighbouring cells (Cai et al., 2026; Setiawan et al., 2026; Thomas and Zurzolo, 2026). Pathological-protein burden is therefore chemically and biologically heterogeneous. A soluble oligomer, a membrane-bound intermediate and a mature fibril may respond quite differently to the same nanomaterial intervention.

Protein accumulation is often framed primarily as a problem of excessive production or aggregation. That account is incomplete. The amount of pathological protein detected in a tissue or cell reflects a dynamic balance between generation, cellular uptake, intracellular transport, degradative processing and re-release into the extracellular environment. Greater uptake can be beneficial when the receiving cell retains sufficient endolysosomal capacity to destroy the internalised cargo. Microglia, for example, may accept aggregates transferred from neurons and process them through comparatively active autophagic and lysosomal pathways (Chakraborty et al., 2026b). More uptake, however, is not necessarily better. Scavenger-receptor-targeted nanoparticles can improve net Aβ clearance even while limiting excessive fibrillar Aβ internalisation, indicating that processing efficiency may be more important than cargo entry alone (Gebril et al., 2024). When the degradative system is already compromised, increased internalisation may instead expand an intracellular pool of incompletely processed protein. This material can remain within endosomal compartments or be redirected towards secretion. Mitochondria-targeted lycopene nanodots improved PINK1/Parkin-related mitophagy and dopaminergic-neuronal survival while favouring secretory efflux of pathogenic α-synuclein (Xia et al., 2023). An apparently favourable intracellular response may therefore coexist with redistribution rather than safe disposal.

This distinction places lysosomal homeostasis at the centre of pathological-protein handling. Here, lysosomal homeostasis refers to the integrated capacity to maintain an acidic lumen, functional hydrolases, cargo delivery and fusion, lipid and ion balance, membrane integrity and terminal substrate degradation (Du et al., 2025; Infante-Tadeo and Barber, 2026). These processes are interdependent, and pathological-protein accumulation can further weaken the degradative system expected to remove it. Microglia and astrocytes may continue to internalise extracellular material despite inadequate acidification, but uptake under these conditions does not ensure complete degradation (Quick et al., 2023; Zeng et al., 2025). Put simply, entry into a lysosome is not the same as lysosomal disposal. Productive clearance requires hydrolysis within a competent compartment without return of biologically active cargo to the extracellular space.

Nanomaterials can act at this organelle level by modifying lysosomal acidity, hydrolase activity, autophagic processing and the local lipid or ion environment (Hao et al., 2025; Li et al., 2026; Arotcarena et al., 2022; Lo C. H. et al., 2026). Their accumulation within lysosomes is not uniformly beneficial. Persistent or poorly tolerated materials may impair fusion or intracellular trafficking, disturb proteostasis or generate ion-mediated oxidative stress after intralysosomal dissolution (Yuan et al., 2021; Liang et al., 2025; Shang et al., 2026; Gupta et al., 2022). Therapeutic value therefore depends not simply on lysosomal entry, but on whether the material improves substrate processing without creating an additional organelle burden.

These observations expose an important limitation in the current nanomedicine literature. A reduction in total protein, intracellular fluorescence, plaque burden or aggregate number does not by itself identify the underlying fate of the pathological substrate. It may reflect reduced synthesis, inhibition of aggregation, physical disassembly, lysosomal degradation or extracellular displacement. Nor does aggregate disruption necessarily mean that the remaining species have lost their seeding competence (Setiawan et al., 2026). This review therefore addresses two linked questions. First, how can nanomaterials increase the proportion of pathological proteins that complete lysosomal degradation? Second, how can they reduce the re-release of incompletely degraded proteins and limit the residual seeding activity of the extracellular material that remains? To answer these questions, we examine the influence of protein conformation and cell type, followed by lysosomal acidification, autophagic transport, lipid processing, membrane integrity and metabolic support. We then consider how failure at these stages redirects cargo towards extracellular vesicles, lysosomal exocytosis or direct intercellular transfer, and whether nanomaterial-mediated reductions in protein burden are accompanied by genuine seed inactivation. Accordingly, this review is organised according to the intracellular fate of pathological proteins and its consequences for extracellular release, rather than nanomaterial composition. The resulting conceptual framework linking pathological-protein handling to lysosomal competence, degradation, re-release and residual seeding is summarised in Figure 1.

**Figure 1:**
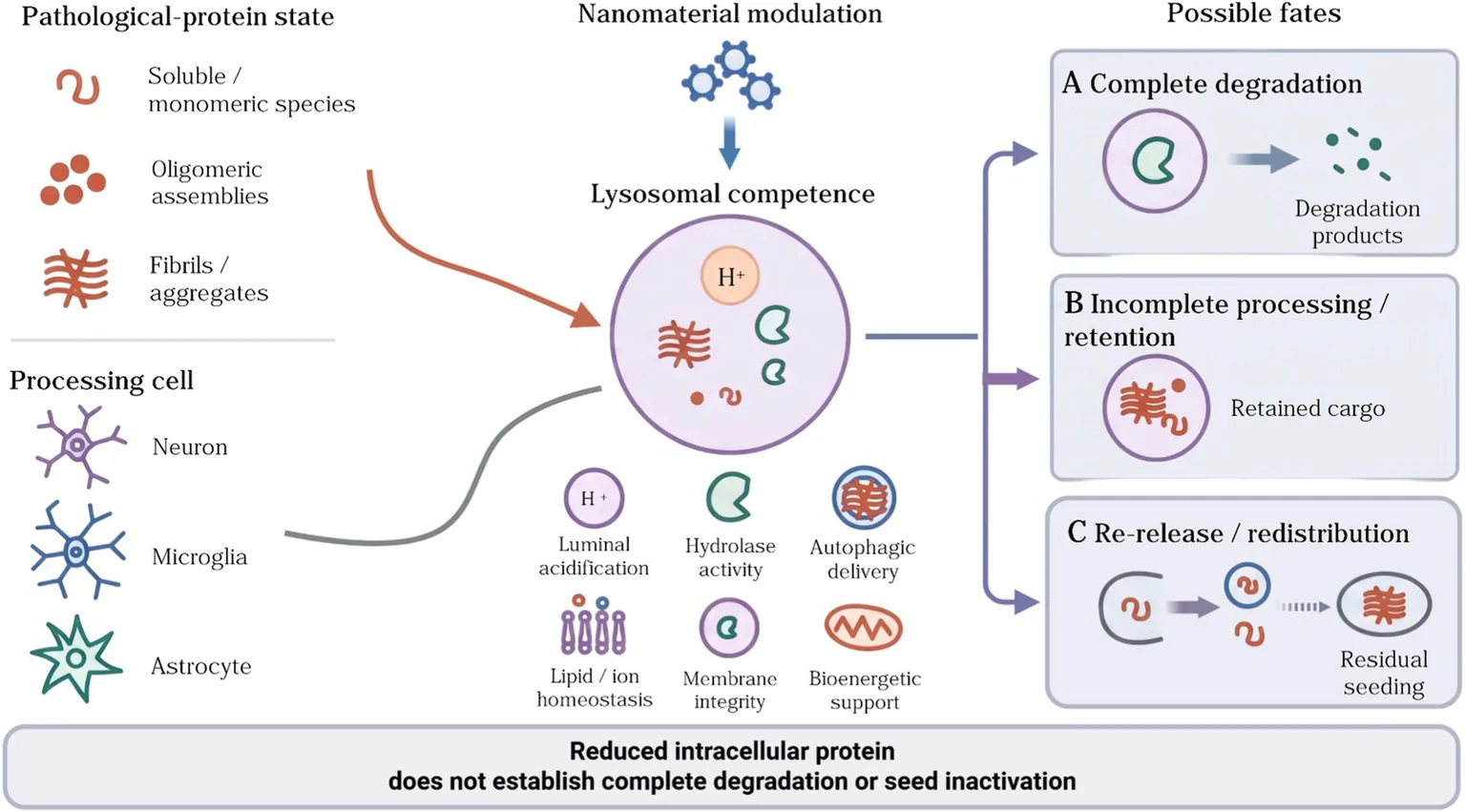
Lysosomal competence shapes pathological-protein degradation, retention and redistribution. Pathological-protein handling depends on substrate conformation and the processing capacity of neurons, microglia and astrocytes. Soluble or monomeric species, oligomeric assemblies and fibrils or aggregates place different demands on the endolysosomal system. Nanomaterials may modulate luminal acidification, hydrolase activity, autophagic delivery, lipid and ion homeostasis, membrane integrity and bioenergetic support. These functions influence three possible cargo fates: (A) complete degradation into breakdown products; (B) incomplete processing with intracellular retention; or (C) re-release or redistribution of incompletely processed material. Released cargo may retain seeding competence and promote aggregation in recipient cells, but release does not itself demonstrate seeding. The dashed connection to residual seeding denotes this conditional outcome. The schematic summarises a conceptual framework across the proteinopathies discussed in the review, rather than a uniform response to all nanomaterials. Reduced intracellular protein abundance alone establishes neither complete degradation nor seed inactivation. Created with BioRender.com

## 2 Literature search and evidence appraisal

This mechanism-focused narrative review was informed primarily by PubMed searches of records published between 31 July 2021 and 30 July 2026. Earlier studies were included selectively when they provided foundational mechanistic evidence, established experimental methods or clarified concepts not adequately addressed in the recent literature. The search strategy was structured around three principal concept blocks: nanomaterials and nanodelivery systems (Concept A), lysosomal and endolysosomal homeostasis (Concept B), and neurodegenerative pathological proteins and their intracellular or extracellular fate (Concept C). The core retrieval strategy combined these three domains using A AND B AND C. Additional combinations were used only where necessary to capture relevant background or mechanistic evidence that was not retrieved by the core search.

After the results from the core and supplementary searches were combined and duplicates removed, 240 potentially relevant records remained. These records were assessed for their relevance to nanomaterial-mediated regulation of lysosomal homeostasis, pathological-protein degradation, re-release or seeding. Records with little or no substantive relevance to the scope of the review were excluded, leaving 157 references, all of which were incorporated into the final synthesis. Original experimental studies were prioritised when they directly examined nanomaterial-mediated changes in lysosomal function or pathological-protein fate, whereas reviews and earlier foundational studies were used mainly to support broader mechanistic, methodological or technological context.

Evidence was grouped into three categories: direct disease-model evidence, mechanistic support and platform extrapolation. Direct disease-model evidence comprised nanomaterial studies performed in neurodegenerative disease models, neural cells, patient-derived cells, organoids or relevant animals that measured pathological-protein processing. Mechanistic support included studies that clarified a relevant lysosomal, lipid, membrane or trafficking mechanism but used non-neural cells, non-neurodegenerative models or interventions other than nanomaterials. Platform extrapolation referred to engineered technologies, such as lysosome-targeting chimeras or other targeted-degradation systems, whose feasibility had been established mainly outside neurodegenerative disease. Such studies were included to identify design opportunities, not as proof of disease-specific efficacy.

The evidential threshold was defined according to the endpoint actually measured. Pathological-protein degradation required direct substrate turnover or loss linked to functional lysosomal or autophagic processing, preferably supported by measurements of luminal pH, hydrolase activity or dynamic autophagic flux. Re-release required measurement of extracellular protein or vesicular cargo rather than vesicle number alone. Reduced seeding activity required a direct biosensor, seed-amplification, recipient-cell aggregation or animal-propagation assay; it was not inferred from lower aggregate abundance. Where studies measured only protein burden, autophagy-related markers, inflammation or behavioural recovery, the conclusions were restricted accordingly.

## 3 Biological and material basis of lysosome-directed nanomedicine

### 3.1 Lysosomal homeostasis, biogenesis and damage responses

Lysosomal capacity is regulated at several levels. Transcription factor EB (TFEB) coordinates lysosomal biogenesis and autophagy-related gene expression, whereas V-ATPase-dependent acidification supports hydrolase maturation and terminal proteolysis (Du et al., 2025; Infante-Tadeo and Barber, 2026; Iyaswamy et al., 2022). These processes depend on continued cargo transport, autophagosome–lysosome fusion, membrane lipid composition, ion flux and cellular bioenergetics. An increase in lysosome number is therefore not equivalent to greater degradative capacity when acidification or downstream flux remains impaired.

Lysosomal damage adds a second constraint. Loss of membrane integrity exposes luminal glycans to the cytosol, where galectins accumulate on injured lysosomes and provide a sensitive marker of membrane damage. Cytosolic cathepsin activity and release of preloaded membrane-impermeant tracers provide complementary evidence of lysosomal membrane permeabilisation (Aits et al., 2015). A transient damage response followed by recovery of acidity represents a different state from persistent leakage and proteolytic failure. Lysosomal swelling or altered LysoTracker signal alone cannot distinguish these outcomes.

### 3.2 Pathological-protein substrates in neurodegenerative proteinopathies

Neurodegenerative proteinopathies present lysosomes with chemically and spatially distinct substrates. Aβ exists in soluble, oligomeric and deposited forms and can occupy extracellular as well as endolysosomal compartments. Tau and α-synuclein are predominantly intracellular proteins but can be released and transferred between cells, whereas polyglutamine-expanded proteins and mutant SOD1 form intracellular aggregation-prone species. Their conformation, membrane association and cellular distribution influence whether an intervention must restrict assembly, enhance intracellular degradation or intercept extracellular cargo (Cai et al., 2026; Setiawan et al., 2026; Thomas and Zurzolo, 2026; Chakraborty et al., 2026b).

The evidence base is not evenly distributed across these substrates. Aβ, tau and α-synuclein account for most lysosome-directed nanomaterial studies. Polyglutamine disease models provide several relevant examples, including lysosomal re-acidification linked to mutant huntingtin degradation, rapamycin-loaded PLGA nanoparticles for mutant ataxin-7 inclusions and ultrasound-responsive removal of neuronal polyglutamine aggregates (Hao et al., 2025; Sarkar et al., 2024; Borbolla-Jiménez et al., 2023). By contrast, direct evidence in SOD1-associated ALS is sparse. PI(3,5)P₂ nanoparticles improved TRPML1 activity, autophagy-related dysfunction and survival in SOD1^G93A^ mice without measuring mutant SOD1 turnover (Tedeschi et al., 2024), whereas CuO nanoparticles induced SOD1 misfolding in a non-neural macrophage model (Gupta et al., 2022). The limited SOD1 coverage therefore reflects a thinner disease-specific evidence base rather than selective exclusion.

### 3.3 Nanomaterial classes, formulations and functional roles

Nanomaterials used in neurodegenerative proteinopathy research span several formulation families. Polymeric systems include PLGA particles, responsive micelles and nanogels; lipid-based platforms include liposomes, self-assembling lipid formulations and lipid nanoparticles. Inorganic and hybrid materials include nanorods, metal or metal-oxide particles, nanozymes and covalent organic frameworks. Carbon-based materials, biomimetic or cell-derived vesicles, DNA nanostructures and multicomponent chimeric platforms provide additional routes for cargo delivery or direct material-mediated activity (Hao et al., 2025; Li et al., 2026; Arotcarena et al., 2022; Lo C. H. et al., 2026; Wang et al., 2025; Xing et al., 2025; Kwatra et al., 2026; Bai et al., 2026; Wu et al., 2026; Li M. et al., 2025; Cui et al., 2024). A single construct may combine more than one class, for example a polymeric core with a biological coating or a targeting ligand.

These materials can function as carriers, as active modulators of lysosomal physiology, or both. Relevant functions include improving brain or cell-specific delivery, re-acidifying lysosomes, restoring hydrolase activity or autophagic flux, binding or disassembling aggregates, modulating lipid and ion balance, and directing selected extracellular or membrane-associated targets towards lysosomal degradation. Composition shapes uptake, intracellular routing, residence time, biodegradation and the risk of organelle stress. It does not, by itself, establish pathological-protein clearance.

## 4 Pathological-protein state and cell-type-specific handling

### 4.1 Aggregation state and susceptibility to degradation

Pathological protein burden is not a uniform degradative target, because molecular form and cellular compartment can shape markedly different clearance outcomes. In Alzheimer’s disease, Aβ may be detected as monomers, oligomers or extracellular deposits, whereas tau pathology encompasses hyperphosphorylated aggregates and neurofibrillary tangles (Cai et al., 2026). This heterogeneity is not merely descriptive. Depletion of KIBRA (WWC1), a trafficking-associated scaffold protein that regulates extracellular-vesicle secretion, reduced Aβ monomers, oligomers and extracellular deposition, yet left intraneuronal APP-CTFβ/Aβ unchanged, indicating that an apparent fall in overall burden may conceal persistent intracellular species (Han et al., 2022). Conversely, TFEB activation by F-SLOH promoted clearance of soluble and insoluble Aβ, phosphorylated tau, APP-CTF15 and PHF-1-reactive tau, representing phosphorylated tau detected by the PHF-1 antibody, while also reducing plaques and tau aggregates (Iyaswamy et al., 2022). These findings accord with the broader view that tau misfolding, post-translational modification, aggregation and seeding are related but distinct pathological states (Setiawan et al., 2026). Likewise, inhibition of Aβ oligomerisation or fibril formation should not be conflated with autophagy-mediated removal of established aggregates (Zamanian et al., 2025).

Aggregate size, membrane association and post-translational state shape both the accessibility of pathological proteins to lysosomes and the integrity of the degradative machinery itself. In cortical neurons, α-synuclein fibrillar polymorphs, Aβ fibrils and Aβ oligomers each reduced microtubule-dependent endosomal transport by approximately twofold, whilst Aβ oligomers increased lysosome number and size but reduced the fraction of motile lysosomes (Chou et al., 2022). Internalised oligomeric Aβ bound ATP6V0C, whereas PHF-1-reactive tau engaged ATP6V1B2; both interactions impaired V-ATPase activity and endolysosomal function (Kim S. H. et al., 2023). Membrane context further modified this relationship: smaller nanoparticles, greater affinity for α-synuclein, sphingosine accumulation and GBA1 deletion favoured detergent-resistant, membrane-associated α-synuclein inclusions (Jiang et al., 2021). Evidence from protein-corona-bearing nanoplastics and APP-compartment models further supports a role for surface assemblies and membrane-associated sorting, although these studies provide indirect mechanistic support (Gopinath et al., 2021; Singh et al., 2025). Aggregate state therefore influences not only degradative accessibility but also the functional capacity of the endolysosomal system, creating a reciprocal relationship between substrate accumulation and lysosomal dysfunction (Thomas and Zurzolo, 2026).

Across proteinopathies, aggregate clearance appears to depend less on a single universal mechanism than on matching the intervention to the physical and cellular state of the substrate. In Alzheimer’s models, TFEB activation by F-SLOH promoted removal of soluble and insoluble Aβ, phosphorylated tau, APP-CTF15 and PHF-1-reactive tau, indicating that several aggregation states can be cleared when autophagy–lysosomal capacity is reinforced (Iyaswamy et al., 2022). PolyQ pathology showed a related, though not identical, response: rapamycin-loaded PLGA nanoparticles cleared mutant ataxin-7 inclusions in a glial model of SCA7 (Borbolla-Jiménez et al., 2023). In synucleinopathies, lysosomal re-acidification enhanced α-synuclein degradation and reduced pathology induced by patient-derived Lewy body extracts (Arotcarena et al., 2022), whereas restoration of GCase activity reduced α-synuclein accumulation in a preformed-fibril model (Kwatra et al., 2026). More refractory fibrils required a different strategy, with released β-synuclein peptides cooperating with HSC70-initiated chaperone-mediated autophagy to eliminate neuronal α-synuclein fibrils (Wu et al., 2025). Acidic liposomes combined with cathepsin B likewise produced greater aggregate removal than pH restoration alone (Jeon et al., 2025). These findings support state-dependent clearance, but not a simple linear rule; structural complexity and uncertain pathogenic species still preclude a genuinely universal approach (Wang D. et al., 2023).

A reduction in pathological protein burden cannot, by itself, distinguish inhibition of aggregate formation from disassembly or degradative clearance. Quercetin reduced Aβ aggregation by 45–60% in preclinical Alzheimer’s models, yet this outcome establishes an anti-aggregation effect rather than lysosomal destruction of pre-existing deposits (Pratap Swain et al., 2026). Similar ambiguity applies to curcumin and berberine, which were reported both to reduce Aβ aggregation and to promote autophagy, without showing that the two effects reflect the same process (Ghoflchi et al., 2026). Flavonols likewise inhibited Aβ oligomerisation and fibril formation whilst separately promoting autophagy-mediated clearance of Aβ aggregates (Zamanian et al., 2025). Nanomaterials make this distinction still more important: negatively charged gold nanoparticles and carbon nanodots limited Aβ or tau fibrillisation, whereas photothermal graphene and gold nanorods disrupted preformed aggregates (Wang Y. H. et al., 2026). Graphene materials may also facilitate deposit clearance, although the route responsible remains insufficiently resolved (Tiwari and Tiwari, 2023). Native PLGA further reduced Aβ levels and deposits through combined effects on Aβ production, aggregation and lysosomal clearance (Karthivashan et al., 2026). Anti-aggregation, disassembly and degradation should therefore be treated as related, but experimentally distinct, therapeutic endpoints.

Lower intracellular pathological-protein burden may reflect degradation, redistribution or both. Protein reduction, extracellular release and residual seeding activity should therefore be treated as separate experimental endpoints.

Collectively, these findings indicate that nanomaterial selection should follow substrate state rather than protein abundance alone. Early-stage assembly may be addressed by interfaces that restrict fibrillisation, established aggregates by physical disassembly or conformation-selective recognition, and persistent intracellular species by transport-coupled lysosomal degradation (Wu et al., 2025; Wang Y. H. et al., 2026; Tiwari and Tiwari, 2023; Zheng et al., 2024). In each case, efficacy still requires evidence that the pathological substrate has been disposed of rather than merely reduced, redistributed or structurally remodelled (Wang D. et al., 2023).

### 4.2 Cell-type-specific uptake and processing by neurons and glia

Pathological-protein handling is distributed across neural cell types rather than executed by a single dominant compartment. Neurons and microglia can both internalise therapeutic nanocarriers and participate in Aβ or α-synuclein clearance, yet their downstream contributions differ markedly. In α-synuclein-exposed co-cultures, neuronal degradative capacity and autophagic flux were impaired, whereas microglia showed higher lysosomal turnover and efficiently degraded aggregates transferred from neurons (Chakraborty et al., 2026b). Likewise, Aβ-responsive nanodrugs entered both neurons and microglia, but the reported clearance benefit was attributed chiefly to enhanced microglial phagocytosis and lysosomal processing (Fan et al., 2022). This division is not absolute: dual-targeted systems restored autophagy-related functions in both cell types (Li et al., 2024b), and lipoprotein-inspired nanocomposites promoted lysosomal Aβ degradation in neurons as well as microglia (Wang H. et al., 2023). Astrocytes contribute differently, by restoring LAMP2-associated autophagy–lysosomal function (Huang et al., 2026) and, through IL-3 signalling, stimulating microglial phagocytosis while supporting neuronal autophagic clearance (Zhang et al., 2024). Selective microglial targeting without neuronal or astrocytic uptake further underscores that cellular entry is experimentally separable from productive degradation (Badr et al., 2024).

Neuronal disposal of pathogenic proteins is constrained less by their initial internalisation than by whether the resulting cargo remains mobile and completes intracellular degradation. In primary cortical neurons, α-synuclein fibrillar polymorphs, Aβ fibrils and Aβ oligomers produced a two-fold decrease in the fraction of fluorescent nanodiamonds transported along microtubules, despite only slightly reducing their interaction with neurons. Aβ oligomers also increased lysosome number and size while decreasing the proportion of moving lysosomes, indicating that organelle expansion may accompany impaired logistics rather than greater degradative capacity (Chou et al., 2022). A similar mismatch emerged after α-synuclein exposure: neuronal lysosomes showed compromised degradation and impaired autophagic flux, whereas inhibition of aggregate clearance increased tunnelling-nanotube-mediated transfer to microglia, which subsequently upregulated autophagy and degraded the transferred aggregates (Chakraborty et al., 2026b). With oAβ, membrane injury and lysosomal exocytosis were followed by extensive endocytosis, accumulation within LAMP1-positive multivesicular bodies or lysosomes, and intercellular propagation through nanotube-like conduits. Importantly, internalised pathological cargo does not share a single fate. Depending on substrate state and cellular processing capacity, it may undergo lysosomal degradation, extracellular release or direct intercellular transfer (Chakraborty et al., 2026b; Han et al., 2022; Dilna et al., 2021). Although nanocomposites and IL-3-based systems can improve neuronal Aβ or α-synuclein clearance (Wang H. et al., 2023; Zhang et al., 2024), efficient neuronal disposal still requires evidence beyond increased uptake or lysosome abundance.

Microglia possess a comparatively strong capacity to internalise and process extracellular pathological cargo, but this advantage reflects functional activity rather than proven differences in lysosome abundance. Their professional phagocytic role encompasses cellular debris, toxic protein aggregates and neuron-derived harmful material, with subsequent clearance requiring both phagocytic and autophagic competence (Quick et al., 2023; Lin et al., 2022). Nanoparticle studies point in the same direction. Polydopamine nanoparticles were efficiently internalised by human microglia and accumulated preferentially in lysosomal compartments (Ceccarelli et al., 2025), while Aβ-responsive formulations enhanced microglial phagocytosis and lysosomal Aβ clearance despite also entering neurons (Fan et al., 2022). Moreover, europium-doped cerium oxide nanoparticles increased CD36 expression alongside Aβ uptake, endolysosomal trafficking and degradation, whilst reducing inflammatory activity (Machhi et al., 2022). Yet these findings do not establish that microglia contain more lysosomes than neurons or astrocytes under comparable conditions. Rather, they support a higher functional degradative reserve whose effectiveness remains dependent on intact lysosomal acidification (Quick et al., 2023), and which can also be shared with neurons under appropriately designed interventions (Wang H. et al., 2023).

Microglial uptake becomes beneficial only when intracellular processing remains competent. Scavenger-receptor-targeted nanoparticles reduced excessive fibrillar Aβ internalisation whilst accelerating lysosomal degradation of material already present, indicating that net clearance can improve without maximising uptake (Gebril et al., 2024). In Parkinson’s disease, limiting RAGE-mediated α-synuclein uptake while increasing mature cathepsin D similarly improved aggregate handling (Zheng et al., 2025). Conversely, silica-coated magnetic nanoparticles caused lysosomal swelling, loss of proteolytic activity, reduced ATP and greater Aβ accumulation in BV2 cells (Shin and Lee, 2024). These contrasting findings show that uptake, intracellular retention and completed degradation are experimentally distinct outcomes.

Astrocytes appear to buffer pathological protein and lipid burden through a combination of direct cargo processing and regulation of neighbouring cells, although the supporting evidence is not equally strong across substrates. Recent work attributes both phagocytic and autophagic activity to astrocytes and links complete degradation to adequately acidified lysosomes; when acidification fails, cellular debris, excess myelin, lipids and toxic protein aggregates accumulate together, with accompanying neuroinflammatory and neurodegenerative consequences (Zeng et al., 2025). Experimental support for restoring this capacity is more limited but consistent. Cy3-labelled PLGA nanoparticles restored lysosomal acidity and cathepsin D activity in stressed astrocytes and reduced chloroquine-associated LC3-II accumulation; cathepsin B activity was also recovered in compromised RPE cells (Li et al., 2026). Uptake varied between adjacent cells and across cell types, while prolonged lysosomal retention was demonstrated in iPS-RPE cells, indicating that exposure to the same platform can differ substantially by cellular context. *In vivo*, Angiopep-2-functionalised liposomes loaded with vorinostat (SAHA@LIPO-ANG2) increased LAMP2 expression in astrocytes while enhancing microglial Aβ phagocytosis, indicating complementary rather than interchangeable roles in Aβ clearance (Huang et al., 2026). Astrocyte-derived IL-3 adds a further layer by promoting microglial phagocytosis of pathological α-synuclein whilst supporting neuronal autophagic clearance (Zhang et al., 2024). Thus, astrocytes are best viewed as conditional regulators and secondary processors of pathological cargo, whose contribution depends critically on preserved lysosomal competence.

Microglial handling of pathological proteins is determined by a shifting functional state, not by a stable M1/M2 identity. In Alzheimer’s disease, elevated microglial miR-17 coincided with defective autophagy, Aβ accumulation and increased inflammatory cytokine production; selective miR-17 inhibition improved autophagy and reduced both Aβ burden and inflammation, showing that clearance and inflammatory control can change together (Badr et al., 2024). Similar coupling was observed in Parkinson’s disease, where restoration of mitochondrial membrane potential, reduced oxidative stress and enhanced autophagy accompanied α-synuclein clearance (Li L. et al., 2025), while TRPV4-targeted treatment combined metabolic reprogramming, sustained lysosomal function and increased phagocytosis (Lo H. et al., 2026). Metabolism itself may therefore be decisive: shifting disease-associated microglia from glycolysis towards oxidative phosphorylation restored surveillance, phagocytosis and autophagy-mediated degradation of Aβ, tau and α-synuclein (Wang D. et al., 2023). Yet inflammatory markers alone do not define clearance competence. Polydopamine nanoparticles lowered ROS, CD40, CD86 and cytokine release without directly measuring pathological-protein degradation (Ceccarelli et al., 2025), and a nanomodulator directed at chronically activated, so-called M1 microglia improved immune regulation but did not establish enhanced aggregate clearance (Wei et al., 2025). Likewise, β-gal-activated senolysis reduced Aβ in aged mice, although the targeted senescent cells were not identified specifically as microglia (Ji et al., 2024). More broadly, microglial states vary with lipid sensing, glycolysis, mitochondrial injury, autophagy failure and innate immune signalling (Xing et al., 2026); excessive α-synuclein uptake may even promote antigen presentation and inflammatory secretion unless degradation is simultaneously restored (Zheng et al., 2025). Microglial clearance is better characterised by measurable phagocytic, metabolic, degradative and secretory functions than by binary labels.

## 5 Nanomaterial regulation of lysosomal degradation of pathological proteins

### 5.1 Restoration of lysosomal acidification and hydrolase activity

Lysosomal acidification is not merely a pH read-out; it is a functional requirement for terminal cargo degradation. The lysosomal lumen is maintained at roughly pH 4.5 through V-ATPase activity, counterion flux, membrane composition and nutrient-sensitive signalling, creating the conditions required for proteolysis and membrane trafficking (Infante-Tadeo and Barber, 2026). Once this environment deteriorates, autophagic and endocytic cargo can still enter the compartment, but degradation becomes inefficient (Du et al., 2025). This distinction is particularly relevant in glia. Microglia may continue to engulf debris and protein aggregates despite impaired acidification (Quick et al., 2023), whilst astrocytes can retain lipids, cellular remnants and toxic proteins when lysosomal acidity is inadequate (Zeng et al., 2025). Lysosomal entry, then, is only a trafficking event. Acidification helps determine whether that event ends in proteolysis. The coordinated lysosomal processes that support productive degradation of internalised cargo are integrated in Figure 2.

**Figure 2:**
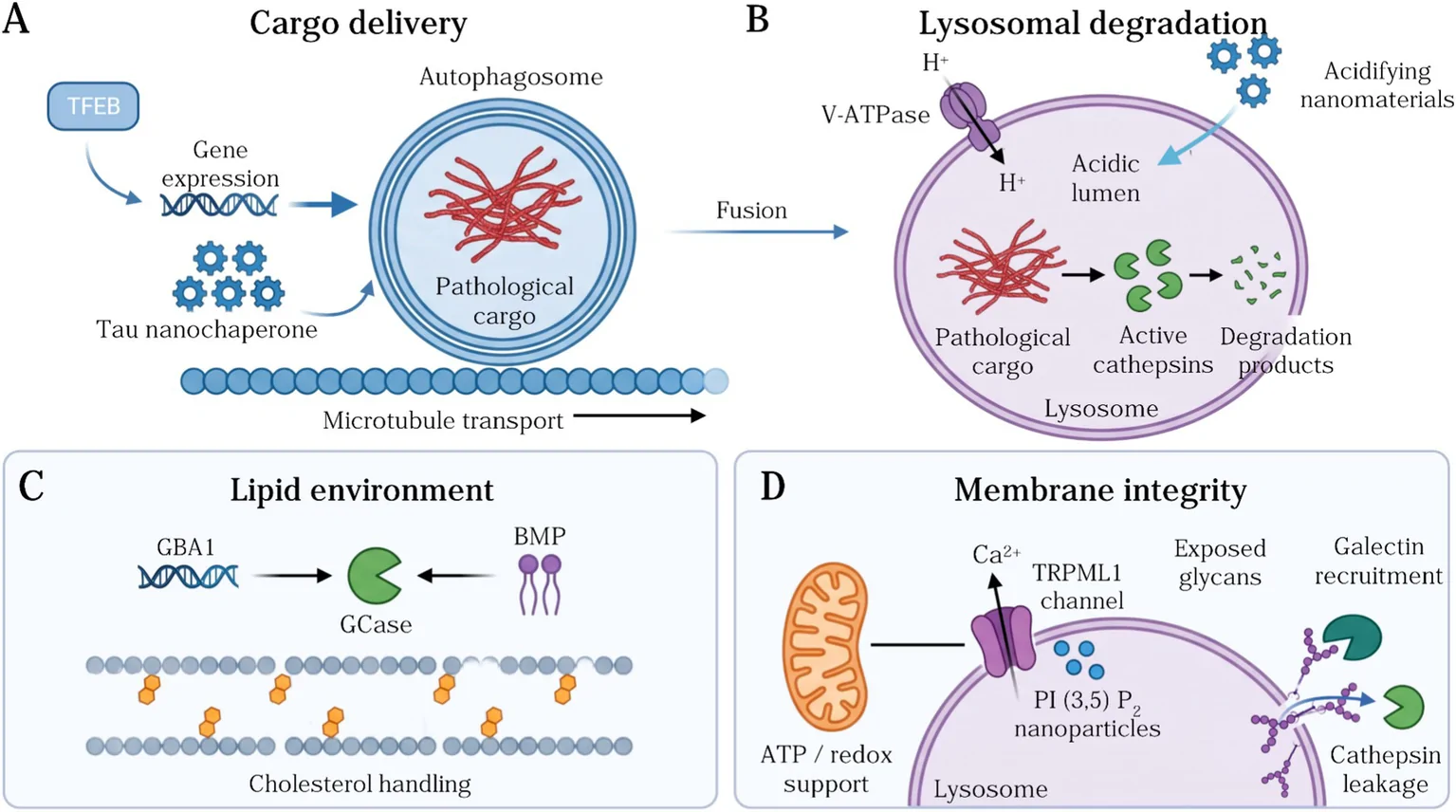
Lysosomal processes and nanomaterial intervention sites governing pathological-protein degradation. **(A)** Cargo delivery. Transcription factor EB (TFEB)-dependent gene expression supports autophagic and lysosomal capacity. The tau nanochaperone illustrates a strategy linking pathological-cargo recognition to microtubule-dependent transport and autophagosome–lysosome fusion. **(B)** Lysosomal degradation. Vacuolar H^+^-ATPase (V-ATPase)-dependent acidification supports cathepsin activity and substrate proteolysis. Acidifying nanomaterials may compensate for inadequate luminal acidity; this does not establish restoration of the initiating V-ATPase defect. **(C)** Lipid environment. GBA1 encodes lysosomal β-glucocerebrosidase (GCase), whose activity is supported by bis(monoacylglycero)phosphate (BMP). Cholesterol handling represents an additional component of lysosomal lipid homeostasis. **(D)** Membrane integrity and functional support. Mitochondrial ATP supply, redox balance and regulated Ca^2+^ release through transient receptor potential mucolipin 1 (TRPML1) contribute to organelle function. Phosphatidylinositol 3,5-bisphosphate [PI(3,5)P₂] nanoparticles illustrate TRPML1 modulation, without establishing direct membrane repair or mutant SOD1 turnover. Membrane damage exposes luminal glycans to cytosolic galectins and permits cathepsin leakage; galectin recruitment and leakage provide complementary indicators of membrane injury. The panels integrate mechanisms and representative interventions from distinct studies, rather than a single experimentally validated treatment sequence. ATP, adenosine triphosphate; SOD1, superoxide dismutase 1. Created with BioRender.com

V-ATPase dysfunction provides a convergent explanation for de-acidification across otherwise distinct proteinopathies, although the initiating lesions differ. In Alzheimer’s disease models, internalised Aβ interacted with ATP6V0C, whereas PHF-1-reactive tau bound ATP6V1B2; both interactions impaired V-ATPase activity and endolysosomal function (Kim S. H. et al., 2023). In microglia exposed to plasma-derived EVs reported as exosomes in patients with Parkinson’s disease, reduced ATP6V1G1 expression was followed by lysosomal swelling, weaker enzyme activity and intracellular α-synuclein accumulation (Li et al., 2024a). GBA1-mutant dopaminergic neurons showed a distinct defect in which constitutive mTORC1 signalling was associated with impaired V-ATPase assembly, reduced lysosomal acidification and lower protease activity. Acidic nanoparticles restored lysosomal pH and improved downstream mitochondrial quality-control measures, although reversal of the upstream molecular defect was not established (Sheshadri et al., 2026). This pattern is more consistent with functional compensation than with direct repair of the initiating lesion.

The consequence of insufficient acidification becomes most apparent at the level of hydrolase maturation and activity. Attapulgite nanorods restored lysosomal pH, increased mature cathepsin D and relieved autophagic blockade, with a parallel increase in mutant huntingtin degradation (Hao et al., 2025). Cy3-labelled PLGA nanoparticles similarly recovered cathepsin B activity and active cathepsin D in lysosomally stressed cells (Li et al., 2026). The reverse pattern is equally informative: defective acidification in a fibroin-deficient silkworm model reduced mature cathepsin L1 and was accompanied by p62, ubiquitinated-protein and aggregate accumulation (Xia et al., 2025). Yet pH is not the sole determinant of enzyme function. In ATP13A2-deficient cells, re-acidification or BMP supplementation restored GCase activity, whereas stored polyamines also interfered with BMP-supported GCase through a partly pH-independent electrostatic mechanism (Samaddar et al., 2025). For this reason, enzyme abundance alone is a weak endpoint. Mature or active hydrolases, substrate-cleavage activity and direct functional assays are more informative (Li X. et al., 2025).

Direct acid delivery offers the clearest test of whether a nanomaterial can alter the lysosomal lumen itself. Succinic-acid-based nucleolipid prodrugs restored neuronal lysosomal acidity after intracellular release, and multimerisation lowered the concentration required to recover an optimal pH (Brouillard et al., 2021; Brouillard et al., 2023). Polymeric systems reached a similar functional endpoint through somewhat less clearly resolved chemistry. PEFSU nanoparticles re-acidified lysosomes and enhanced autophagic clearance of A30P α-synuclein in cellular and Drosophila models (Lo C. H. et al., 2026), whilst acidic nanoparticles improved α-synuclein degradation and reduced pathology in mice challenged with patient-derived Lewy body extracts (Arotcarena et al., 2022). These studies support the principle of lysosomal re-acidification, but they also illustrate an important distinction: a material may demonstrably restore pH without its intralysosomal acid-release mechanism being fully defined.

Acidifying capacity matters only if the material reaches the appropriate brain region, cell population and lysosomal compartment. Mesenchymal-stem-cell-derived nanovesicle coatings increased the brain penetration of PLGA nanoparticles and were associated with restored lysosomal pH, lower p62 and reduced APP and phosphorylated tau (Gu et al., 2026). Gas6-engineered stem-cell membranes provided more selective access to pathological regions and microglia, where Aβ clearance and lysosomal acidification improved (Sha et al., 2025). Even so, organelle delivery remained heterogeneous. Cy3-labelled PLGA nanoparticles achieved high lysosomal localisation in iPS-RPE cells, but uptake varied between adjacent cells and across cell types (Li et al., 2026). Brain entry and lysosomal localisation therefore represent distinct levels of exposure, neither of which establishes uniform delivery to the relevant intracellular compartment.

Across proteinopathies, the strongest evidence is obtained when re-acidification is linked to hydrolase recovery and direct substrate loss. This combination has been demonstrated for mutant huntingtin (Hao et al., 2025) and α-synuclein (Arotcarena et al., 2022; Lo C. H. et al., 2026), whereas several Alzheimer’s disease platforms have mainly shown broader reductions in Aβ, APP fragments or phosphorylated tau (Bai et al., 2026; Gu et al., 2026; Sha et al., 2025). Further gains may require more than pH correction. Co-delivery of an acidic lysosome-targeted formulation with cathepsin B produced a synergistic improvement in aggregate clearance (Jeon et al., 2025), whilst COF-HQ combined acidification rescue with copper chelation, suppression of Aβ–Cu^2+^ aggregation and ROS scavenging (Bai et al., 2026). PI(3,5)P₂ nanoparticles improved TRPML1 activity and autophagy without demonstrating direct pH rescue (Tedeschi et al., 2024). Taken together, these studies argue for coordinated restoration of the acidic milieu, hydrolase function and the lipid- or ion-dependent processes that support degradation.

A fall in lysosomal pH is an important pharmacodynamic signal, but terminal disposal requires additional functional evidence. Probe position must be resolved carefully, since DNA nanodevices have detected a juxta-lysosomal acidic layer distinct from the luminal environment (Zhang Y. et al., 2026). Functional assessment should combine lysosomal pH with V-ATPase assembly, mature or active cathepsins, p62 turnover, inhibitor-controlled autophagic flux and pathological-protein turnover (Li et al., 2026; Sheshadri et al., 2026). Ultimately, the benefit of re-acidification depends on whether autophagic cargo can still reach the lysosome and complete degradation.

### 5.2 Enhancement of autophagic transport and lysosomal degradation

Therapeutically useful autophagy is defined by completed flux, not by the appearance of more autophagic structures. LC3-II accumulation can indicate increased autophagosome formation, but it can also reflect a block in downstream degradation. A more defensible interpretation combines LC3 with p62 or ubiquitinated-substrate turnover, tandem fluorescent reporters and lysosomal-inhibitor controls. In motor-neuron-like cells, mCherry–GFP–LC3 imaging, LC3-II/LC3-I measurements and electron microscopy together supported increased flux after protocell treatment (Gonzalez Porras et al., 2023). By contrast, Atg8-PE rose together with p62 and ubiquitinated proteins in the fibroin-deficient silkworm, and bafilomycin A1 produced no further p62 accumulation, indicating a terminal block (Xia et al., 2025). Silica-coated magnetic nanoparticles and food-grade nanoparticles offer disease-relevant counterexamples: autophagy-related markers increased or persisted whilst lysosomal function and Aβ or phosphorylated tau clearance deteriorated (Shang et al., 2026; Shin and Lee, 2024). Even a reduction in LC3 may be compatible with benefit when excessive or stalled mitophagy is corrected (Mongkolpobsin et al., 2023). The direction of LC3 change alone is therefore insufficient to establish whether autophagic flux has increased or become obstructed.

Upstream signalling becomes therapeutically persuasive only when downstream processing also recovers. Nanocarrier-delivered simvastatin activated AMPK–ULK1-related signalling and reduced Aβ plaques, but the reported endpoints primarily demonstrate pathway engagement rather than completed flux (Afify et al., 2026). Stronger evidence comes from a Prussian blue nanozyme that linked AMPK/ULK1 activation and mTOR inhibition to restored autophagic flux and tau aggregate clearance (Wu et al., 2026). Cell-selective activation provides another route. Photothermal stimulation of microglial TRPV1 engaged Ca^2+^/CaMKK2/AMPK/mTOR signalling, increased ATG5-dependent autophagy and promoted α-synuclein phagocytosis and degradation (Yuan et al., 2022). DRD3-targeted chitosan nanoparticles similarly combined SIRT1-related signalling with LAMP2 expression, increased lysosomal capacity and pSer129-α-synuclein clearance (Biswal et al., 2026). Together, these studies indicate that upstream pathway activation becomes mechanistically meaningful only when it coincides with preserved lysosomal capacity and measurable loss of the relevant pathological substrate.

Nanodelivery is particularly useful when it corrects poor stability, restricted tissue exposure or inadequate intracellular release of established autophagy modulators. ROS-responsive, neuron-targeted micelles released rapamycin within affected regions, restored autophagic flux and reduced intracellular Aβ and hyperphosphorylated tau in 3 × Tg-AD mice (Xu et al., 2022). The importance of payload release was demonstrated by trehalose nanogels: only the releasing formulation, rather than non-releasing gels or free trehalose, reduced α-synuclein-induced Lewy body-like inclusions (Maruf et al., 2025). By comparison, transferrin-decorated rapamycin carriers improved brain delivery and reduced Aβ, but autophagy was inferred mainly from LC3 and mTOR-associated measurements rather than direct dynamic-flux analysis (Khonsari et al., 2022). Natural-product nanoformulations may likewise improve solubility, chemical stability and sustained exposure. However, reports involving diosgenin, quercetin and EGCG largely support enhanced pharmacological feasibility, anti-aggregation activity or autophagy-associated responses rather than completed lysosomal degradation, because dynamic flux and pathological-substrate turnover were not consistently assessed (Abidi et al., 2024; Acıkara et al., 2022; Al Amin et al., 2025).

Nucleic-acid delivery allows autophagy to be adjusted in defined cell populations rather than amplified across the brain. Mannose-coated lipid nanoparticles lowered miR-17 selectively in microglia, improved autophagy and reduced Aβ burden in 5XFAD mice (Badr et al., 2024). Other platforms combined reduced protein production with enhanced disposal. Lactoferrin-functionalised lipid nanoparticles co-delivering α-mangostin and BACE1 siRNA suppressed BACE1 whilst promoting microglial uptake and autophagic degradation of Aβ (Xu et al., 2025); an intranasal system combined BACE1 siRNA, rapamycin and the Aβ-binding peptide KLVFF to reduce production, inhibit aggregation and promote clearance (Yang et al., 2022). In Parkinson’s disease, ROS-responsive carbonised polymer dots delivered plasmid DNA, reduced SNCA expression and restored autophagic flux through TFEB nuclear translocation (Zhai et al., 2025). These multifunctional platforms act at several stages of the pathological-protein pathway, although the relative contributions of reduced protein synthesis, inhibited aggregation and enhanced lysosomal degradation remain difficult to separate.

The capacity of the terminal compartment must expand alongside autophagic input. TFEB activation by F-SLOH increased lysosomal biogenesis and autophagy–lysosome degradation, with reductions in APP fragments, Aβ and phosphorylated tau (Iyaswamy et al., 2022). A dual neuron–microglia regulator paired rapamycin-driven autophagy induction with gypenoside XVII-mediated lysosomal improvement, thereby addressing both cargo input and terminal processing (Li et al., 2024b). The opposite effect was produced by polystyrene nanoplastics, which activated TSC2–mTOR signalling, inhibited TFEB nuclear translocation and disrupted autophagosome–lysosome fusion (Liang et al., 2025). Selective recruitment of pathological substrates may limit the burden created by indiscriminate amplification of global autophagic input. Microglia-derived nanovesicles synchronised macroautophagy and chaperone-mediated autophagy (Li M. et al., 2025), whilst ultrasound-responsive nanodrugs induced robust flux and removed neuronal polyglutamine aggregates (Sarkar et al., 2024). Targeted autophagy technologies extend this principle beyond general pathway activation. Autophagosome-tethering compounds (ATTECs) recruit selected cargo to LC3-associated autophagosomes; autophagy-targeting chimeras (AUTACs) promote selective autophagic removal of tagged substrates; and AUTOTACs couple target recognition to p62-dependent autophagic machinery. Chaperone-mediated autophagy (CMA)-based strategies provide a related route for selective lysosomal delivery. These approaches can, in principle, direct proteins, aggregates or organelles towards degradation, although much of the platform evidence remains outside neurodegenerative models (Zhou et al., 2025).

Neurons add a further bottleneck: cargo-bearing autophagosomes must travel over long distances before they can fuse with lysosomes. A tailored Nano-dredger co-delivered siROCK2 and lithospermic acid B, improved microtubule function and ATP availability, and restored retrograde transport of autophagosomes for lysosomal degradation (Meng et al., 2025). A pathogenic-tau-selective nanochaperone—an engineered nanoplatform that binds pathological tau while supporting microtubule stability and autophagic trafficking—approached the same problem from the substrate side. Bound tau was sequestered into autophagosomes and transported towards lysosomes, enhancing autophagosome–lysosome fusion and tau clearance (Xu et al., 2024). Together, these studies show that neuronal autophagic clearance depends not only on pathway activation, but also on the physical transport of sequestered cargo to degradatively competent lysosomes.

Autophagy nevertheless operates within a wider proteostatic and metabolic network. Nanomaterials may alter the ubiquitin–proteasome system (Ai et al., 2025), whilst ROS can either trigger adaptive autophagy or overwhelm it, depending on intensity and duration (Jin et al., 2026). Mitochondrial injury can impair lysosomal function and restrict autophagic flux while simultaneously favouring extracellular-vesicle release (Kou et al., 2024). These interactions converge on a common constraint: increasing autophagic input without preserving lipid handling, membrane integrity and cellular energetics may deliver more cargo to lysosomes without ensuring terminal degradation. Productive autophagy therefore depends on the wider organelle environment in which lysosomal processing occurs.

### 5.3 Lysosomal lipid processing and the sustainability of pathological-protein clearance

The degradative burden placed on a lysosome is not limited to the protein aggregate itself. Lipids, lipid-bound metals and amphiphilic molecules share the same membranes and intralysosomal environment, altering enzyme access, membrane rigidity and cargo organisation (Dufourc, 2024). This is especially relevant in glia, where the TREM2–APOE lipid-sensing axis intersects with immunometabolic reprogramming and autophagy failure (Xing et al., 2026). Greater uptake may therefore add cargo without improving net clearance if the receiving cell cannot mobilise or metabolise the accompanying lipids. Acidic PLGA nanoparticles improved lysosomal acidification, autophagy and aggregate removal in macrophages with defective debris and lipid clearance, providing useful mechanistic support even though the model was not neurodegenerative (Zhang et al., 2023). A lipid carrier that delivers an autophagy modulator does not, by that property alone, restore lysosomal lipid processing. Transferrin-decorated lipid carriers reduced Aβ and altered mTOR-related signalling, but did not demonstrate restoration of cholesterol transport, lipid export or lysosomal lipid turnover (Khonsari et al., 2022).

The GBA1–GCase axis shows how the local lipid environment can actively constrain proteolysis. In ATP13A2-deficient cells and patient-derived neurons, lysosomal polyamine accumulation was accompanied by secondary BMP and glucosylsphingosine storage and reduced GCase activity. Re-acidification or BMP supplementation restored enzyme activity, yet polyamines also interfered with BMP-supported GCase through a partly pH-independent electrostatic mechanism (Samaddar et al., 2025). A related limitation is evident in GBA1-mutant dopaminergic neurons. Acidic nanoparticles restored lysosomal pH and improved downstream mitochondrial quality-control measures, but direct clearance of the associated lipid burden was not demonstrated (Sheshadri et al., 2026). Restoration of lysosomal acidity therefore does not, on its own, establish normalisation of lipid handling.

Gene delivery reaches the same problem from a different direction. Polymeric nanoparticles carrying human GBA1 produced sustained transgene expression in the substantia nigra, reduced α-synuclein accumulation and preserved dopaminergic neurons in an α-synuclein preformed fibril (PFF) model (Kwatra et al., 2026). Because glucosylceramide, glucosylsphingosine and sphingosine were not reported, sphingolipid handling remains unresolved despite the reduction in α-synuclein burden. Nanomaterial–protein interactions may further alter this relationship. Particles with affinity for α-synuclein promoted detergent-resistant, membrane-rich inclusions when endolysosomal processing was impaired, and GBA1 deletion increased susceptibility in association with higher intracellular sphingosine (Jiang et al., 2021). The evidence therefore supports a directional chain from disturbed lipid conditions to weaker proteolysis and greater aggregation vulnerability. It does not establish the reverse claim that α-synuclein accumulation was itself responsible for the GCase defect.

The therapeutic implication is that lipid correction and acidification should not be treated as unrelated processes. In ATP13A2-deficient cells, pH and BMP each modified GCase activity (Samaddar et al., 2025), whereas acidic PLGA improved the degradation of debris, lipids and protein aggregates in macrophages with defective clearance (Zhang et al., 2023). Although these models represent different pathological contexts and levels of relevance to neurodegenerative disease, they converge on the same constraint: recovery of degradative function may remain incomplete when the lipid burden of the compartment persists.

Cholesterol mobilisation offers a second route to relieve lipid stress. Hydroxypropyl-β-cyclodextrin and related cyclodextrins can mobilise cholesterol and reduce pathological lipid accumulation, and engineered derivatives may also influence autophagic flux and mitochondrial function (Pirvu et al., 2025; Rejili et al., 2026). Much of this literature, though, concerns platform development or lysosomal-storage disorders rather than direct nanomaterial-mediated degradation of neurodegenerative proteins. The glial context is equally important. Reducing Ndst1 expression lowered intracellular lipid accumulation in wild-type mouse astrocytes and in astrocytes expressing humanised APOE3 or APOE4, consistent with a broader influence of heparan-sulfate signalling on autophagy, mitochondrial function and lipid metabolism (Schultheis et al., 2024). Together with the microglial lipid–metabolic coupling outlined above, these findings indicate that durable clearance depends on the metabolic state of the processing cell. Cholesterol-directed materials may improve membrane properties or substrate availability, but they are unlikely to sustain clearance when cellular metabolism remains compromised.

Lipid droplets are useful accompanying read-outs because they capture a part of the cellular burden that protein measurements miss. Copper-chelating carbon dots reduced copper-induced protein aggregation together with lipid-droplet accumulation, cholesteryl esters and triglycerides (Bathla et al., 2026). These results show that protein and lipid stress can be modified in parallel, but they do not prove that either pool underwent lysosomal degradation. Organelle resolution can sharpen the interpretation. A tetrahedral-DNA FRET probe detected lysosomal PLD3 localisation in normal neurons and greater mitochondrial overlap in Alzheimer’s disease neurons (Wen et al., 2025), illustrating how a nominally lysosomal protein may be redistributed when organelle homeostasis is disturbed. Lipid-droplet area or number is most informative when paired with lipid-species measurements and subcellular localisation.

Disease context should also guide which lipid endpoint is prioritised. In synucleinopathy, GCase activity and sphingolipid species are central because they intersect directly with α-synuclein handling (Kwatra et al., 2026; Jiang et al., 2021; Sheshadri et al., 2026; Samaddar et al., 2025). In Alzheimer’s disease, cholesterol distribution, APOE-associated lipid transport and glial lipid droplets may be more informative (Xing et al., 2026; Pirvu et al., 2025; Schultheis et al., 2024). These are overlapping rather than identical problems. A single “lipid homeostasis” marker would blur that difference. Nanomaterial studies should therefore select lipid measurements according to the proteinopathy and the target cell, while retaining a common core of lysosomal pH, hydrolase function and pathological-protein turnover.

Evaluation of sustained pathological-protein clearance requires parallel assessment of lysosomal lipid handling and protein-substrate fate. Lysosomal pH, V-ATPase assembly and GCase or other hydrolase activity establish organelle competence. Glucosylceramide, glucosylsphingosine, free and esterified cholesterol, triglycerides, cholesterol efflux and lipid-droplet turnover define the accompanying lipid burden. Protein half-life, aggregate load, degradation products and undegraded intracellular residue then show what happened to the pathological substrate. Individual studies currently cover only parts of this chain: GBA1 delivery measured α-synuclein and neuronal outcomes without full sphingolipid profiling (Kwatra et al., 2026), whereas carbon dots quantified lipid changes without establishing terminal lysosomal disposal (Bathla et al., 2026). The strongest evidence for sustained clearance will come when lower pathological-protein burden is accompanied by less lipid storage and demonstrably restored lipid-processing dynamics.

### 5.4 Lysosomal membrane integrity, bioenergetic support and nanomaterial-induced organelle stress

Lysosomal membrane integrity preserves luminal acidity, confines hydrolases and separates degradative chemistry from the cytosol. Once the membrane becomes permeable, cathepsins and other lysosomal contents can escape, producing anything from a limited, potentially recoverable lesion to lysosome-dependent cell death. Several nanomaterial studies report changes compatible with lysosomal injury, including ferritinophagy, iron accumulation and lipid peroxidation after silver-nanoparticle exposure (Zhai et al., 2022), impaired lysosomal activity and cholesterol trafficking after silica exposure (Wang H. et al., 2026), and lysosome-associated copper release with proteostatic stress, mitochondrial damage and Cu/Zn SOD1 misfolding in macrophages (Gupta et al., 2022). These findings do not, however, prove lysosomal membrane permeabilisation. Direct assessment requires evidence such as galectin recruitment to damaged lysosomes, cytosolic cathepsin activity or release of preloaded membrane-impermeant tracers (Aits et al., 2015). Organelle stress and lysosomal membrane rupture are therefore related but experimentally distinct outcomes.

A time course is also essential. A transient galectin response followed by restored acidity and lysosomal function would indicate a different biological state from persistent leakage with cytosolic cathepsin activity. The current nanomaterial studies rarely make that distinction. Without it, apparently similar changes in lysosomal size, LysoTracker signal or cathepsin expression may represent adaptation, membrane injury or terminal failure.

Ca^2+^ signalling may determine whether lysosomal dysfunction remains reversible, although the therapeutic evidence is still incomplete. Nanoparticle delivery of PI(3,5)P₂ stabilised TRPML1 opening, prolonged intracellular Ca^2+^ signalling, improved autophagy and extended survival in SOD1^G93A^ mice (Tedeschi et al., 2024). These findings support lysosomal ion-channel modulation in an ALS model, but the study did not establish direct turnover of mutant SOD1 or membrane repair. Excess Ca^2+^ can have the opposite effect. Glutamate-driven influx was associated with ROS generation, mitochondrial depolarisation and disordered mitophagy (Mongkolpobsin et al., 2023), whilst extrasynaptic GluN2B signalling has been proposed to disrupt autophagy through Ca^2+^-linked mitochondrial dysfunction (Roy et al., 2025). The relevant objective is therefore controlled lysosomal signalling, not a general increase in intracellular Ca^2+^. Direct measures of membrane recovery or removal of damaged lysosomes would be needed before TRPML1 activation could be described as a repair strategy.

Bioenergetic support is another enabling condition rather than a stand-alone mechanism of lysosomal rescue. Naringenin nanocrystals preserved mitochondrial membrane potential, ATP production and redox balance in rotenone-injured SH-SY5Y cells (Giradkar et al., 2024). Mitochondrial positioning and transport also depend on ATP, Ca^2+^ and motor-adaptor systems that distribute energy along neuronal processes (Jain et al., 2023). These observations make biological sense, particularly in long neuronal projections, but the current evidence does not directly show that restoring ATP alone re-establishes lysosomal acidification, repairs lysosomal membranes or completes pathological-protein degradation. Mitochondrial outcomes should therefore be interpreted as support for organelle homeostasis unless a study also measures lysosomal function and substrate fate.

Mitochondrial injury may also alter pathological-protein handling by promoting the intercellular redistribution of damaged organelles and protein aggregates. In neuronal and microglial systems, α-synuclein-associated mitochondrial damage promoted the transfer of both α-synuclein and damaged mitochondria from neurons to microglia (Chakraborty et al., 2026a), linking bioenergetic failure to intercellular cargo redistribution. Mitochondrial dysfunction can therefore restrict intracellular degradative capacity while increasing the cargo burden imposed on recipient cells. Nanomelatonin and brain-targeted nanogels have improved mitophagy or mitochondrial biogenesis in Parkinsonian models (Biswal et al., 2024; Qin et al., 2026), whereas silver-nanoparticle exposure increased PINK1 and Parkin but suppressed PGC-1α, producing disturbed mitophagy rather than recovery (Chang et al., 2022). As with LC3, changes in mitophagy-associated markers must be linked to completed organelle turnover.

A stronger mechanistic case emerges when redox control is coupled to restored lysosomal processing. COF-HQ combined Cu^2+^ chelation and ROS scavenging with recovery of Aβ-impaired lysosomal acidification and microglial Aβ clearance (Bai et al., 2026). RLA-rHDL@ANG similarly linked Aβ binding and aggregate disruption to lysosomal transport and degradation in neurons and microglia (Wang H. et al., 2023). The Prussian blue nanozyme and ROS-responsive rapamycin micelles joined antioxidant effects to restored autophagic flux and reductions in tau, Aβ or hyperphosphorylated tau (Wu et al., 2026; Xu et al., 2022). These platforms do more than improve the metabolic background, but even here the contribution of redox correction should be separated from direct aggregate binding, autophagy activation and lysosomal delivery. Natural-antioxidant formulations that report only ROS, mitochondrial or behavioural improvement provide indirect evidence of lysosomal benefit unless lysosomal function and pathological-substrate fate are assessed concurrently.

Conversely, several exposure studies identify conditions under which nanomaterials impair rather than support lysosomal clearance. Polystyrene nanoplastics disrupted TFEB signalling and autophagosome–lysosome fusion (Liang et al., 2025), whilst repeated exposure to food-grade nanoparticles reduced neuronal lysosomal function and impaired Aβ and phosphorylated-tau clearance (Shang et al., 2026). Silica nanoparticles similarly aggravated α-synuclein aggregation in association with defective autophagic processing (Yuan et al., 2021). Metal-containing materials introduce an additional mechanism of injury through intracellular ion mobilisation. Silver nanoparticles activated NCOA4-associated ferritinophagy, increased labile iron and lipid peroxidation, and reduced GPX4, with smaller particles producing greater toxicity (Zhai et al., 2022). Pharmacological rescue with ferrostatin-1 or deferoxamine further linked these effects to iron-dependent injury (Zhai et al., 2024). These findings identify disrupted lysosomal fusion, ion release, ferritinophagy and lipid peroxidation as plausible mechanisms by which nanomaterials can weaken proteostatic clearance. Detailed assessment of particle size, dose, repeated exposure and regulated cell-death pathways is required to establish the translational safety of individual formulations.

Material persistence further complicates the balance between organelle-localised therapy and lysosomal burden. Cy3-labelled PLGA particles remained detectable in lysosomes for at least 28 days whilst restoring acidity and cathepsin activity (Li et al., 2026). Such persistence may prolong therapeutic exposure, but its consequences under repeated dosing and after treatment withdrawal remain unresolved. The biological meaning of prolonged residence therefore depends on material degradation, ion release, lysosomal swelling, membrane integrity and post-treatment recovery. Stable pathological-protein clearance requires preservation of the membrane and metabolic conditions that sustain lysosomal function throughout treatment.

## 6 Pathological-protein re-release and residual seeding activity

### 6.1 Cellular routes of pathological-protein re-release

Pathological-protein re-release is not a separate event that begins only after degradation has failed. It emerges from the same endosomal, lysosomal and autophagic network that is expected to clear the cargo. Multivesicular bodies (MVBs) provide the clearest example of this competition. In KIBRA-deficient 5XFAD mice and APP-overexpressing cells, APP-CTFβ/Aβ-containing MVBs accumulated and fused more frequently with lysosomes as extracellular-vesicle secretion declined; inhibition of lysosomal function restored extracellular vesicle (EV) secretion (Han et al., 2022). This is unusually direct evidence that degradation and export can draw from the same intracellular pool. Other findings suggest that recycling-endosome-derived secretory compartments and mitochondrial–lysosomal stress may influence the same balance (Singh et al., 2025; Kou et al., 2024). Because these observations derive mainly from non-neural models or review-level synthesis, they provide mechanistic support but less direct evidence for pathological-protein re-release in neural systems. Endosomal identity matters, but lysosomal competence determines whether sorting ends in degradation or export.

Lysosomal insufficiency can enlarge the pool available for vesicular diversion, but intracellular accumulation and release must be measured separately. Plasma-derived EVs described as exosomes in the source study reduced microglial ATP6V1G1 expression, impaired lysosomal acidification and enzyme activity, and increased intracellular α-synuclein (Li et al., 2024a). These data demonstrate defective degradation, not increased secretion by the recipient microglia. More direct evidence comes from astrocytes: α-synuclein deposition increased astrocyte-derived EV release, and α-synuclein-carrying EVs were elevated in patients with Parkinson’s disease (Wang P. et al., 2023). In an Alzheimer-type model, brain-derived EVs contained increased tau, phosphorylated tau and Aβ, whereas serum EVs reproduced only part of the tissue pattern (de la Monte et al., 2025). Lysosomal dysfunction may therefore favour EV-associated export, but the inference is strongest when vesicle output and pathological cargo are measured in the same system. A rise in intracellular protein creates the opportunity for release; it is not the release event itself.

EV number alone is a poor measure of pathological output. Concentration, size, cellular origin, cargo per vesicle and recipient-cell activity can move in different directions. FTLD studies, for example, reported altered vesicle size and complement or cathepsin D cargo even when the concentration of plasma small extracellular vesicles (sEVs) did not increase (Bellini et al., 2022a; Bellini et al., 2022b). These biomarker findings demonstrate that EV concentration alone does not capture changes in vesicle size, cellular origin or cargo composition. EVs can also carry protective material: miR-106b-containing vesicles altered neuronal autophagy (Bai et al., 2021), miR-6780-5p-enriched exosomes reduced polyQ expression (Chen et al., 2023), and small heat-shock proteins may provide proteostatic support (Van den Broek et al., 2021). The term “exosome” should therefore be reserved for vesicles whose endosomal origin is adequately demonstrated, and EV studies should report separation, characterisation, release, uptake and functional testing in line with current field standards (Welsh et al., 2024). The decisive read-out is therefore not particle abundance alone, but the amount, molecular state and biological activity of pathological cargo delivered to recipient cells.

Lysosomal exocytosis creates a second exit route, particularly when the plasma membrane has been damaged. Ca^2+^ entry can trigger lysosome–plasma-membrane fusion as part of rapid membrane resealing (Reddy et al., 2001). Repair is then coupled to rapid endocytosis of the damaged membrane (Idone et al., 2008), aided by extracellular release of lysosomal acid sphingomyelinase (Tam et al., 2010). Oligomeric Aβ links this general repair mechanism to neurodegenerative protein transfer. In differentiated SH-SY5Y cells, oAβ damaged the plasma membrane, evoked Ca^2+^-dependent lysosomal exocytosis and was followed by extensive compensatory endocytosis. Internalised oAβ accumulated in LAMP1-positive MVBs and lysosomes, whilst PAK1-dependent actin remodelling generated tunnelling nanotube (TNT)-like conduits through which oAβ moved between cells (Dilna et al., 2021). A membrane-saving response can therefore alter trafficking in ways that redistribute pathological cargo. It should not be described as extracellular lysosomal degradation unless substrate destruction outside the cell is directly shown.

Autophagy-associated clearance can also coexist with secretory displacement. Mitochondria-targeted lycopene nanodots improved PINK1/Parkin-related mitophagy and dopaminergic-neuronal survival but favoured secretory efflux of pathogenic α-synuclein (Xia et al., 2023). This finding shows that intracellular benefit may coexist with increased extracellular exposure to pathological protein. By contrast, miR-6780-5p-enriched exosomes increased autophagy and reduced polyQ expression without measuring protein export (Chen et al., 2023). Reduced intracellular staining after nanointervention may therefore reflect degradation, outward movement or both. An experiment must collect the extracellular fraction to tell the difference.

These release routes are not mutually exclusive. MVB sorting, lysosomal exocytosis, EV production and TNT formation can all be altered by the same upstream stressors, including impaired acidification, mitochondrial injury and cytoskeletal remodelling. Discrimination among these release routes requires pathway-resolved analysis rather than attribution of all extracellular protein to a single secretory mechanism. Pharmacological or genetic interruption of Rab-dependent EV release, lysosomal exocytosis or actin-dependent TNT formation, combined with fractionated analysis of soluble and vesicular protein, would help identify the dominant route. The question is not simply how much protein leaves the donor cell, but how it leaves and what happens next.

Tunnelling nanotubes provide a direct route for cargo transfer between connected cells. In human neuronal and microglial systems, α-synuclein aggregates induced mitochondrial damage, mtDNA release and cGAS–STING–NF-κB–IRF3 activation. The resulting actin remodelling promoted TNT-like transfer of α-synuclein and damaged mitochondria from neurons to microglia, where the transferred mitochondria underwent lysosome-mediated degradation (Chakraborty et al., 2026a). Microglia also displayed greater lysosomal turnover than neurons, while inhibition of neuronal autophagic clearance increased TNT-mediated α-synuclein transfer to microglia (Chakraborty et al., 2026b). Oligomeric Aβ produced a related sequence involving plasma-membrane damage, lysosomal exocytosis, compensatory endocytosis and TNT-like propagation (Dilna et al., 2021). Such transfer may unload stressed neurons onto cells with greater degradative reserve, but it also provides a route for pathological spread (Thomas and Zurzolo, 2026). Donor-cell protein loss, extracellular cargo and recipient-cell consequences must therefore be measured separately to distinguish compensatory redistribution from propagation.

### 6.2 Seeding activity of released pathological proteins and nanomaterial intervention

Seeding-related evidence can be separated into increasingly specific experimental endpoints rather than treated as a general study-quality score. Tier 1 records a reduction in total pathological-protein abundance, which may reflect lower synthesis, altered distribution or degradation. Tier 2 requires a reduction in inclusions, aggregates or fibrils and therefore establishes structural improvement but not loss of biological activity. Tier 3 requires direct evidence of lower templating activity in a FRET-based biosensor, seed-amplification assay or recipient-cell aggregation system. Tier 4 extends this evidence to reduced spread or recipient pathology in an animal inoculation or brain-propagation model. Tau biosensors, α-synuclein RT-QuIC and *in vivo* Aβ inoculation assays illustrate the assays used at Tiers 3–4 (Meyer-Luehmann et al., 2006; Groveman et al., 2018; Holmes et al., 2014). Most nanomaterial studies reach Tiers 1–2; lower protein or aggregate burden should therefore not be interpreted as direct seed inactivation. The principal routes of pathological-protein transfer and the four-tier framework for evaluating residual seeding are summarised in Figure 3.

**Figure 3:**
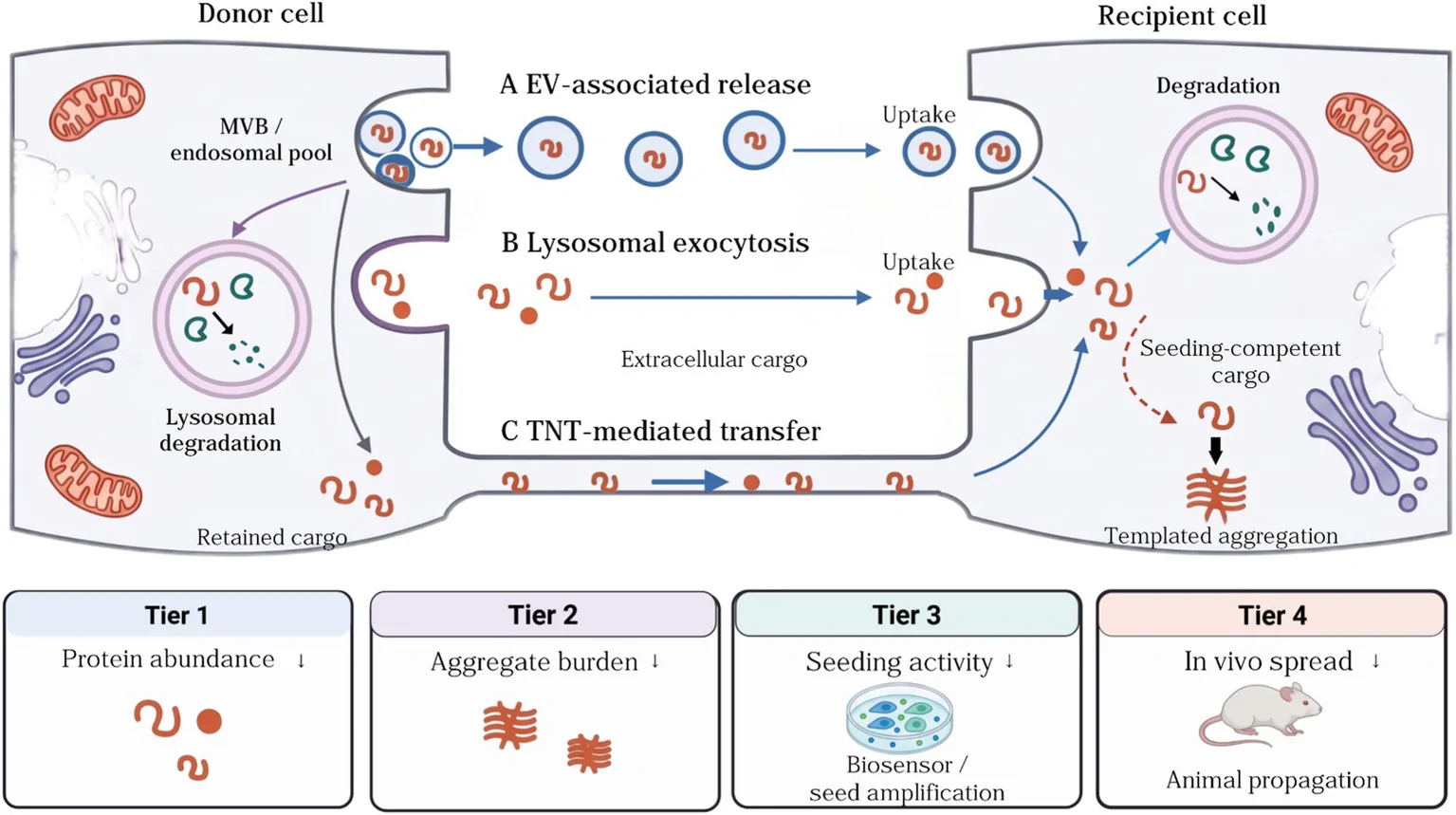
Pathological-protein transfer routes and endpoint-specific evidence for reduced seeding. Pathological cargo in donor cells may undergo lysosomal degradation, remain incompletely processed or enter intercellular transfer routes. (A) Extracellular vesicle (EV)-associated release from the endosomal or multivesicular-body (MVB) pool, followed by recipient-cell uptake. (B) Lysosomal exocytosis, through which cargo is released into the extracellular space and may subsequently be internalised by recipient cells. (C) Tunnelling nanotube (TNT)-mediated transfer between connected cells. Recipient cells may degrade transferred material; templated aggregation is a conditional outcome when seeding-competent cargo persists, as indicated by the dashed branch. The lower strip distinguishes four operational evidence tiers: reduced total pathological-protein abundance (Tier 1), reduced aggregate or fibril burden (Tier 2), directly reduced seeding activity in biosensor, seed-amplification or recipient-cell aggregation assays (Tier 3), and reduced *in vivo* spread or recipient pathology (Tier 4). These tiers describe endpoint specificity, not an overall study-quality score or an obligatory biological sequence. Most nanomaterial evidence reviewed here remains concentrated at Tiers 1–2. Donor-cell protein loss, released or transferred cargo and recipient-cell consequences therefore require separate assessment. Created with BioRender.com

Across the current evidence base, nanomaterial studies provide substantially stronger support for reductions in protein abundance, fibril formation and aggregate burden than for direct loss of seeding activity. Evidence at Tiers 3 and 4 remains scarce, particularly where seed inactivation must be linked to reduced release from donor cells and diminished pathology in recipients.

Nanomaterial interfaces can alter the early stages of amyloid assembly, but their effects depend on surface chemistry and the protein–lipid environment. EGCG-based approaches inhibit Aβ aggregation, reduce tau hyperphosphorylation and improve autophagy-related outcomes, yet these findings do not establish reduced transmissible activity (Al Amin et al., 2025). Negatively charged gold nanoparticles, carbon nanodots and graphene-based materials can bind amyloidogenic proteins and restrict fibrillisation or higher-order assembly (Wang Y. H. et al., 2026; Tiwari and Tiwari, 2023). Tannin behaviour shows why simple anti-aggregation language can be misleading: amphiphilic molecules redistribute among proteins, lipids and membranes according to competing interactions (Dufourc, 2024). Adsorption to a nanomaterial surface may alter local concentration, orientation and aggregate architecture without eliminating the underlying protein species. These platforms therefore provide Tier 1–2 evidence of altered assembly. They do not yet resolve whether nucleation, fibril elongation or the abundance of soluble, highly active intermediates has changed.

Disassembly of a mature fibril is likewise a structural endpoint, not proof that seeding has been extinguished. Black phosphorus nanosheets bind α-synuclein fibrils, trigger disaggregation and activate autophagy, with accompanying improvements in cellular and animal outcomes (He et al., 2024). Photothermal graphene or gold-nanorod systems can disrupt preformed aggregates and reduce plaque-related pathology (Wang Y. H. et al., 2026). What remains after disruption is less clear. Smaller assemblies may be more readily degraded, but they may also retain or redistribute biological activity. Fibril loss, subsequent lysosomal clearance and seed inactivation should therefore be measured separately. At minimum, the post-treatment supernatant or released protein fraction should be recovered and tested rather than assumed to be innocuous. Until this has been done, the appropriate conclusion is structural remodelling with improved clearance-related outcomes, not confirmed suppression of propagation.

Selective recognition of pathological conformations offers a more controlled route because aggregate binding is coupled to a degradative pathway. A customised nanochaperone recognised pathogenic tau, stabilised microtubules and enabled tau-containing autophagosomes to reach and fuse with lysosomes, thereby reducing tau burden and improving disease-related outcomes in mice (Xu et al., 2024). Gold-nanoshell ATB nanoparticles used externally triggered release of β-synuclein peptides and HSC70-associated chaperone-mediated autophagy to eliminate neuronal α-synuclein fibrils (Wu et al., 2025). Both platforms go beyond non-specific disassembly and reach Tier 2, with a mechanistically plausible route towards Tier 3. Their mechanistic strength lies in connecting recognition, intracellular transport and lysosomal processing. Neither study, however, directly tested whether residual tau or α-synuclein had lost seeding activity, nor whether material released from treated cells induced less aggregation in recipients.

Targeted degradation may also reduce the intracellular reservoir from which releasable seeds arise. Autophagy-based technologies can recruit individual proteins, aggregates and organelles to lysosomal processing, although neurological application remains constrained by brain delivery, cell-type dependence and the difficulty of distinguishing pathogenic from physiological protein pools (Zhou et al., 2025; Lu et al., 2026). In disease-relevant models, trehalose-releasing nanogels reduced α-synuclein-induced Lewy body-like inclusions (Maruf et al., 2025), whilst ultrasound-responsive micelles induced strong autophagic flux and removed neuronal polyglutamine aggregates (Sarkar et al., 2024). These are meaningful Tier 2 outcomes. They do not show that extracellular release or recipient-cell seeding has fallen. Nor do behavioural or survival improvements resolve this question, because the donor cell may benefit whilst bioactive material remains outside it. The proposed benefit—shrinking the pool available for later propagation—remains mechanistically plausible but experimentally untested.

Lysosome-targeting chimeras (LYTACs) extend targeted degradation to extracellular and membrane-associated proteins by coupling target recognition to receptor-mediated lysosomal trafficking. Programmable DNA LYTACs and related bifunctional nanoplatforms have degraded EGFR or PD-L1 in tumour systems (Xing et al., 2025; Cui et al., 2024; Cheng et al., 2025). In a neurological setting, a polyvalent endocytosis-triggering chimera (endoTAC) enhanced RAGE internalisation and lysosomal degradation while serving as a simvastatin-delivery platform in an Alzheimer’s disease model (Wang et al., 2025). These systems establish engineering feasibility, but RAGE is a disease-associated receptor rather than a seeding-competent protein species, and direct lysosomal elimination of Aβ, tau or α-synuclein seeds has not yet been demonstrated. A disease-specific construct would still need to show selective capture of a pathogenic conformer, lysosomal destruction and reduced activity in a direct seeding assay.

Among the studies reviewed here, none simultaneously assessed restored lysosomal function, complete degradation of a defined pathological substrate, reduced extracellular release and loss of seeding activity within a single experimental framework. Resolving this gap will require intracellular substrate turnover to be linked directly to the quantity and molecular state of released protein. Soluble and EV-associated fractions should be analysed separately, and recovered extracellular material should be tested using protein-specific biosensors, seed-amplification assays or recipient-cell aggregation systems. Claims of interrupted propagation require a further step: reduced pathology after animal inoculation or brain-spread analysis. Donor-cell rescue and recipient-cell safety should therefore be evaluated within the same experimental sequence. Until these endpoints are connected, reductions in total protein or visible aggregates support conclusions at Tiers 1 or 2, but not direct seed inactivation. Representative nanomaterial studies are compared in Table 1 according to material class, pathological substrate, lysosomal mechanism, directly measured substrate fate and evidence category. Extracellular release and seeding are shown separately because neither can be inferred from a reduction in intracellular protein or aggregate burden.

**Table 1:** Representative nanomaterial interventions mapped to material class, lysosomal function and pathological-protein fate.

Platform	Material/Formulation class	Model/Substrate	Target cell/Context	Primary mechanistic axis	Lysosomal/Flux endpoint	Direct substrate fate	Extracellular release assessed	Seeding activity assessed	Evidence class
Acidic nanoparticles (Arotcarena et al., 2022)	Acidic PLGA nanoparticles	α-synuclein; PD models	neuronal / *in vivo*	lysosomal re-acidification	pH / lysosomal function	α-syn degradation / pathology ↓	No	No	Direct disease-model
PEFSU nanoparticles (Lo C. H. et al., 2026)	PEFSU-based polymeric acidic nanoparticles	A30P α-syn; cellular + Drosophila	neuronal context	re-acidification + autophagy	lysosomal pH / autophagic clearance	α-syn degradation ↑	No	No	Direct disease-model
Attapulgite nanorods (Hao et al., 2025)	Inorganic mineral nanorods	mutant huntingtin	disease-relevant cellular model	acidification + hydrolase maturation	pH / mature cathepsin D / flux	mutant huntingtin degradation ↑	No	No	Direct disease-model
Cy3-PLGA nanoparticles (Li et al., 2026)	PLGA polymeric nanoparticles	lysosomal stress model	astrocytic / cellular	re-acidification + hydrolase recovery	pH / cathepsin B & D / autophagic processing	fate evidence is mechanistic rather than disease-specific	No	No	Mechanistic support
Polymeric GBA1 delivery (Kwatra et al., 2026)	Polymeric gene-delivery nanoparticles	α-syn PFF model	nigral neuronal context	GCase restoration	lysosomal enzyme axis	α-syn accumulation ↓	No	No	Direct disease-model
ATB nanoparticles (Wu et al., 2025)	TRPV1-targeted gold nanoshell / β-synuclein peptide nanosystem	neuronal α-syn fibrils; PD model	dopaminergic neuronal / in vivo	β-synuclein release + HSC70-initiated CMA	CMA-linked lysosomal processing	α-synuclein fibril elimination	No	No	Direct disease-model
Sequence-targeted lycopene nanodots (Xia et al., 2023)	TPP-functionalised recombinant H-ferritin nanocages carrying lycopene	pathological α-synuclein; MPTP PD models	dopaminergic neuronal / in vivo	mitochondrial targeting + PINK1/Parkin mitophagy	mitophagy-associated processing	pathogenic α-synuclein redirected towards secretory efflux	Yes—increased	No	Direct disease-model
COF-HQ (Bai et al., 2026)	Covalent organic framework nano-chelator	Aβ; AD-related model	microglial context	acidification + Cu^2+^ chelation + ROS control	lysosomal acidification / clearance function	Aβ clearance ↑	No	No	Direct disease-model
Prussian blue nanozyme (Wu et al., 2026)	Prussian blue inorganic nanozyme	tau pathology	disease-relevant neural model	AMPK/ULK1 activation + mTOR inhibition	dynamic autophagic flux	tau aggregate clearance ↑	No	No	Direct disease-model
Ultrasound-responsive nanodrug (Sarkar et al., 2024)	Sonosensitiser-loaded polymer micelles	toxic polyglutamine aggregates	neuronal cellular model	ultrasound-triggered singlet oxygen + autophagy induction	increased autophagic flux	polyglutamine aggregate clearance ↑	No	No	Direct disease-model
Pathogenic-tau nanochaperone (Xu et al., 2024)	Customised pathogenic-tau-selective nanochaperone	pathogenic tau; mouse model	neuronal / in vivo	conformation recognition + microtubule transport	autophagosome transport / lysosomal fusion	tau clearance ↑	No	No	Direct disease-model
Intelligent modular DNA LYTAC (IMTAC) (Cui et al., 2024)	Programmable circular-DNA-origami LYTAC nanodevice	EGFR / PD-L1; tumour model	non-neurological platform model	receptor-mediated lysosomal targeting	lysosomal internalisation / degradation	EGFR and PD-L1 degradation	No	No	Platform extrapolation

The studies shown are representative rather than exhaustive and are organised by material class, pathological substrate, cellular context, lysosomal mechanism and the endpoints directly assessed. Extracellular release and seeding measurements were not criteria for inclusion in the table. The lycopene-nanodot study is included because pathological α-synuclein secretory efflux was directly assessed, illustrating that an improvement in intracellular or cellular outcomes may coexist with increased extracellular redistribution. The platform-extrapolation example demonstrates lysosome-targeting engineering feasibility outside neurodegenerative disease and should not be interpreted as evidence of disease-specific efficacy. Seeding activity is marked only where directly tested using functional seeding assays; the representative nanomaterial studies shown here did not directly establish reduced residual seeding activity.

## 7 Design principles and translational evaluation of nanomaterials targeting lysosomal homeostasis

### 7.1 Hierarchical delivery to the brain, disease-relevant cells and subcellular targets

Brain-targeted nanomedicine should be evaluated as a sequence of distribution events rather than by a single measurement of brain uptake. A nanomaterial must first cross or bypass the blood–brain barrier (BBB), reach the affected brain region, enter the cell population responsible for handling the pathological substrate and, finally, arrive at the intracellular compartment in which its payload is intended to act. Failure at any stage can render an apparently successful formulation biologically ineffective. This is particularly important for lysosome-regulating platforms. Endothelial uptake does not establish transcytosis, parenchymal exposure does not prove delivery to the relevant neural cell, and cellular internalisation does not demonstrate access to a functional lysosome (Ardah et al., 2026; Gao et al., 2025; Liu et al., 2026). Targeted degradation and autophagy-modulating technologies face the same hierarchy, with additional constraints imposed by limited brain bioavailability, cell-type dependence and the intracellular location of the intended target (Lu et al., 2026; Seo et al., 2026). Brain accumulation is therefore an initial exposure measure, not a complete delivery endpoint.

Receptor-mediated transcytosis is useful only when receptor binding is coupled to productive endothelial sorting and release on the parenchymal side. Angiopep-2-containing carriers demonstrated LRP1-associated uptake and barrier passage in separate experimental systems, but regional and cellular distribution after crossing remained incompletely resolved (Chakravarty et al., 2026). PepH3-modified carriers showed active transcytosis in rat and human blood–brain barrier models and preferential trafficking through the endoplasmic reticulum and Golgi apparatus rather than lysosomes (Szecskó et al., 2025). This intracellular route may reduce endothelial lysosomal sequestration and favour transcytosis, but endothelial passage still needs to be distinguished from delivery to diseased neural tissue. Glucose-derived carbon dots provide a more complete distribution example: their localisation was mapped across brain regions, neural cell populations and intracellular compartments, revealing near-exclusive neuronal uptake and residence within late endosomal–lysosomal compartments (Seven et al., 2025). Claims of successful brain targeting are therefore strongest when endothelial transport, tissue distribution, cellular identity and subcellular localisation are assessed together.

Alternative routes can reduce reliance on systemic blood–brain barrier transcytosis, but they do not remove the need to establish final distribution. Intranasal systems have used olfactory-epithelial binding or lactoferrin-associated transport to deliver BACE1 siRNA, rapamycin and related payloads (Xu et al., 2025; Yang et al., 2022). Extracellular-vesicle-based and biomimetic carriers may provide membrane compatibility, cargo protection and some degree of innate tissue preference (Nouri et al., 2024; Tiwari et al., 2025; Zhang et al., 2025). Their transport route and final recipient cell, however, are not always clear. Microglia-derived nanovesicles accumulated in inflammatory Alzheimer’s disease regions but were subsequently internalised by neurons (Li M. et al., 2025). Focused ultrasound may offer spatial control over barrier permeability and local delivery (Bano et al., 2025), yet barrier opening alone does not identify which cells or organelles receive the material. Irrespective of the route used, meaningful delivery requires confirmation of tissue, cellular and organellar exposure.

Cellular targeting should reflect both the pathological state and the function expected from the recipient cell. In microglia, the objective is not simply to maximise aggregate uptake. Scavenger-receptor-targeted nanoparticles reduced excessive fibrillar Aβ internalisation whilst accelerating lysosomal degradation of material already present, thereby improving net clearance and reducing inflammatory activation (Gebril et al., 2024). This distinction is important because microglial uptake may become detrimental when lysosomal processing is inadequate. Other systems have used Gas6-engineered stem-cell membranes, mannose-associated receptors or activation-responsive ligands to increase delivery to microglia in diseased regions (Sha et al., 2025; Zhu et al., 2026). Lineage targeting nevertheless remains relatively broad. Microglial states differ in lipid handling, metabolic activity, autophagic competence and secretory behaviour. A receptor associated with a defined pathological state may therefore provide greater functional precision than a marker expressed across the entire microglial population.

Neuron- and astrocyte-directed systems require a similarly explicit match between payload and intracellular function. Neuronal delivery may be appropriate for intracellular tau, α-synuclein, polyglutamine-expanded proteins or gene-regulatory payloads. A neuron-specific polymer system delivered C9orf72 siRNA after endosomal escape (Garcia-Guerra et al., 2023), whilst targeted protocells enhanced autophagic flux in motor-neuron-like cells (Gonzalez Porras et al., 2023). Tau-inhibitory peptides required both cellular uptake and subsequent release from the carrier to avoid organellar entrapment (Reich et al., 2024). Evidence for astrocyte-directed delivery remains less extensive, although Angiopep-2-modified nanoparticles have been associated with increased LAMP2 and recovery of astrocytic autophagy–lysosome function (Huang et al., 2026). Broad entry into neurons, astrocytes and microglia is not inherently advantageous. Each cell population must receive a cargo suited to the pathological substrate it contains or processes and to the pathway that the intervention is intended to modify.

Subcellular localisation is not a generic design advantage; its value depends on the payload’s site of action. Materials intended to restore lysosomal acidity, supply hydrolases or promote lysosomal degradation require sufficient access to the endolysosomal compartment. Nucleolipid formulations restored neuronal lysosomal acidity (Brouillard et al., 2021), whilst nanovesicle-coated PLGA particles combined improved brain entry with recovery of lysosomal pH and reduced p62 (Gu et al., 2026). Scavenger-receptor-targeted nanoparticles and endocytosis-triggering chimeras likewise relied on lysosomal delivery to degrade fibrillar Aβ or RAGE (Gebril et al., 2024; Wang et al., 2025). The opposite applies to cargos that must act in the cytosol, mitochondria or nucleus. pH-sensitive polymers enabled endosomal escape of C9orf72 siRNA (Garcia-Guerra et al., 2023); charge-reversing carriers released catalase into the cytosol (Hu et al., 2026); other systems escaped lysosomes before directing peptides or redox-active components towards mitochondria (Qian et al., 2022; Wang L. et al., 2026) or plasmid DNA towards the nucleus (Zhai et al., 2025). High lysosomal localisation and efficient endosomal escape are therefore opposing routing objectives selected according to payload function.

Residence time further modifies the relationship between organelle targeting and therapeutic function. Lysosome-directed materials need sufficient residence to alter luminal pH, hydrolase activity or substrate processing, but prolonged retention may impose an additional burden on the same compartment. Therapeutic residence should therefore be distinguished from irreversible accumulation by assessing material degradation, lysosomal function during exposure and recovery after treatment cessation. For cargos designed to leave the endolysosomal pathway, the fractions that remain sequestered and those successfully released should be quantified separately.

Stimulus-responsive systems are most useful when they order successive delivery events rather than merely adding complexity. Aβ-responsive carriers have coupled tissue entry to local cargo release (Fan et al., 2022), whilst β-galactosidase- or redox-sensitive systems have restricted activation to senescent or oxidatively stressed cells (Ji et al., 2024; Zhai et al., 2025). More elaborate platforms separate extracellular and intracellular functions. One component may support barrier passage or metal chelation, followed by cathepsin-triggered separation, lysosomal escape or organelle-specific delivery (Wang L. et al., 2026; Liu et al., 2022; Mu et al., 2025). Biomimetic coatings can divide similar responsibilities between the membrane and the core. Mesenchymal-stem-cell-derived nanovesicles increased brain penetration, whereas the PLGA component restored lysosomal function (Gu et al., 2026); Gas6-engineered membranes improved microglial targeting around a drug-loaded core (Sha et al., 2025). Biological provenance does not guarantee targeting fidelity, however. Membrane proteins may be masked or inactivated during coating, and the final recipient cell may differ from the membrane’s cell of origin (Li M. et al., 2025; Zhu et al., 2026). Each structural component should therefore solve a distinct delivery bottleneck and contribute a measurable function.

Delivery studies should report the distribution of both the carrier and its payload, because release, degradation or protein-corona formation may cause the two to follow different trajectories. Regional brain concentration should therefore be paired with cell-type- and organelle-resolved measurements, together with the proportion of intact and degraded material at each level. Long-term disposition is equally important. Initial blood–brain barrier permeability, ligand anchoring or cellular uptake does not establish clearance of the carrier, persistence of targeting function or behaviour after repeated administration (Zhu et al., 2026). A complete delivery profile should define where the material and payload accumulate, how long they remain detectable, whether they retain their intended activity and whether the receiving organelle continues to function during and after treatment.

### 7.2 Mechanistic efficacy, material safety and clinical translation

Therapeutic efficacy should be judged by the fate of the pathological protein rather than by phenotypic rescue or isolated pathway markers. MOMB–AgNPs improved behaviour, reduced inflammatory changes and increased LC3β and antioxidant-related measures in P301S mice, yet the reported outcomes did not establish tau turnover, degradation products or loss of seeding activity (Elbermawy et al., 2025). Patient-derived cortical organoids showed another form of divergence: STB-MP reduced Aβ plaques without suppressing phosphorylated tau (Kim N. G. et al., 2023). Native PLGA lowered APP-related products and Aβ in mouse and patient-derived neurons, but also altered gene expression, leaving the mechanism of protein reduction unresolved (Wu et al., 2022). These outcomes remain biologically relevant, but the strength of the conclusion must match the endpoint. Behavioural improvement demonstrates functional benefit; it does not reveal whether the pathogenic substrate was degraded, structurally remodelled or redistributed.

A minimum mechanistic efficacy panel should combine organelle function, dynamic pathway activity and direct substrate fate. Lysosomal pH should be measured alongside V-ATPase assembly or activity and mature or active hydrolases. Autophagic processing requires inhibitor-controlled flux, tandem fluorescent reporters or equivalent dynamic methods rather than LC3-II alone. The pathological substrate should then be followed through protein half-life, pulse–chase analysis, degradation products, aggregate turnover or another direct measure of substrate loss. The amount of undegraded protein remaining inside the cell and the amount released outside it should be reported separately. Studies that combine lysosomal re-acidification, active cathepsins and improved autophagic processing provide several components of this framework (Li et al., 2026), whilst astrocytic α-synuclein studies illustrate how lysosomal function and extracellular-vesicle release can be examined in the same system (Wang P. et al., 2023). Direct measurements of protein turnover remain necessary even when inflammation, ROS or intracellular fluorescence are reduced.

The experimental pipeline should progress across models rather than relying on one system to reproduce the full complexity of neurodegenerative disease. Primary neural cells are suitable for assessing uptake, subcellular localisation, organelle function and substrate turnover. Patient-derived induced pluripotent stem-cell (iPSC) neurons add human genetic and disease context, whilst cortical organoids permit concurrent evaluation of Aβ- and tau-related phenotypes (Kim N. G. et al., 2023; Wu et al., 2022). Neuron–microglia–astrocyte co-cultures are needed when cell-to-cell transfer, selective uptake or compensatory degradation is central to the hypothesis. Patient-derived pathological proteins or seeds can determine whether a platform remains effective against clinically relevant conformations. Animal models are then required for regional distribution, repeated dosing, behavioural outcomes and network propagation. The model should be selected according to the claim: a monoculture can demonstrate lysosomal acidification, but it cannot establish reduced brain-wide spread.

Extracellular release and residual seeding activity require an evaluation arm distinct from intracellular degradation. Soluble protein, EV-associated cargo and material transferred through alternative routes should be separated wherever feasible. EV concentration, size, cellular origin and pathological cargo may change independently and therefore require separate analysis (Wang P. et al., 2023; de la Monte et al., 2025; Bellini et al., 2022b). EV terminology, isolation, characterisation, release and uptake should follow current field standards (Welsh et al., 2024). In accordance with the four-tier framework defined in Section 6.2, reductions in total protein or visible aggregates do not establish loss of seeding activity. Released material should be tested directly using protein-specific biosensors, seed-amplification methods or recipient-cell aggregation assays, with animal inoculation or brain-propagation models reserved for claims of interrupted *in vivo* spread (Meyer-Luehmann et al., 2006; Groveman et al., 2018; Holmes et al., 2014). Evidence is strongest when donor-cell substrate degradation, extracellular cargo and recipient-cell seeding are measured within the same experimental sequence.

Material safety is an organelle-level property as well as a whole-cell outcome. Nanoparticle-induced lysosomal dysfunction is increasingly recognised as a recurrent intracellular mechanism of nanotoxicity, although its extent varies with material composition, physicochemical properties and intracellular persistence (Uzhytchak et al., 2023). Toxicological exposure models are not dose-equivalent to therapeutic formulations, but they reveal failure modes that may become relevant during repeated organelle exposure. At therapeutic doses, the pertinent risks include lysosomal congestion, impaired fusion, reduced hydrolase activity, lipid accumulation and aggravation of the underlying proteinopathy.

Safety evaluation should distinguish particle effects from released ions and from specific regulated cell-death pathways. Ferroptosis is characterised by iron-dependent phospholipid peroxidation and failure of antioxidant defences, with iron metabolism, GPX4 and system Xc^−^ among its principal regulatory axes (Han et al., 2024; Yasam et al., 2026). These background mechanisms do not establish ferroptosis in an individual nanomaterial study. Direct attribution requires relevant molecular changes and, preferably, pharmacological or genetic rescue. Silver nanoparticles activated NCOA4-associated ferritinophagy, blocked autophagic flux and increased intracellular iron in a particle-size-dependent manner (Zhai et al., 2022). Rescue with ferrostatin-1 or deferoxamine reduced iron accumulation, neuronal injury and cognitive impairment in related models, strengthening the link to ferroptotic injury (Zhai et al., 2024). Copper-containing materials require similar caution. CuO nanoparticles accumulated and dissolved within macrophage lysosomes, and copper release was accompanied by proteostatic and mitochondrial injury (Gupta et al., 2022). Broader evidence links disturbed copper homeostasis to copper-dependent cell-death pathways (Zhang S. X. et al., 2026), but cuproptosis should not be assigned without demonstrating copper accumulation, mitochondrial target engagement and pathway-specific effects. Particle toxicity, ion release, ferroptosis, cuproptosis and non-specific oxidative injury should remain experimentally distinct categories.

Organelle safety should be measured directly rather than inferred from cell viability alone. Lysosomal pH, hydrolase activity, autophagosome–lysosome fusion and lipid trafficking should be assessed alongside lysosomal size and number. Suspected lysosomal membrane permeabilisation requires galectin puncta, cytosolic cathepsin activity or tracer-release assays rather than LysoTracker loss alone (Aits et al., 2015). Lipid-related safety endpoints should be selected according to the proteinopathy. GCase activity and sphingolipid species are particularly relevant to synucleinopathy, whereas cholesterol distribution, APOE-associated lipid transport and lipid-droplet turnover may be more informative in Alzheimer’s disease. Material residence, biodegradation and recovery after withdrawal should also be recorded. A formulation that restores lysosomal pH during short exposure may still be unsuitable if repeated administration causes persistent swelling, impaired fusion or incomplete material clearance.

Repeated-dose studies are essential because the same characteristics that support local efficacy may generate cumulative toxicity. Long lysosomal residence can prolong acidification or substrate exposure, but repeated administration may increase intracellular material burden, alter organelle distribution or reduce the capacity to process subsequent doses. Therapeutic studies should therefore report tissue and cellular accumulation after each dose, clearance intervals, changes in particle size or chemical composition after intracellular processing and evidence of organelle recovery before redosing. Safety follow-up should extend beyond the period of behavioural improvement. Delayed lysosomal dysfunction, lipid accumulation, membrane injury or inflammatory activation may not be apparent during short efficacy studies.

Translational development requires an integrated benefit–risk programme rather than isolated efficacy and toxicology studies. Formulation batches used across these studies should be manufactured and characterised under comparable, predefined specifications for particle size, morphology, surface chemistry, payload loading, release kinetics, colloidal stability and biological activity. Autophagy-modulating nanomedicines continue to face challenges in structural reproducibility, scalable production and demonstration of an advantage over conventional treatment (Romero and Morilla, 2021). Biomimetic anchoring may preserve nanoparticle and membrane-protein activity, but high anchoring efficiency and initial BBB permeability do not establish batch consistency, storage stability or long-term disposition (Zhu et al., 2026). Natural-product nanocarriers may improve exposure, yet impurities, variable composition, pharmacokinetic uncertainty and limited human validation remain important constraints (He et al., 2026; Lai et al., 2025; Lehoczki et al., 2025). Additional architectural complexity is justified only when each component contributes a measurable and non-redundant function.

Clinical translation will also require biologically informed stratification, although this principle remains largely prospective. Protein conformation, target-cell state, disease stage and the dominant lysosomal or lipid abnormality may influence whether a platform promotes degradation, leaves the substrate unchanged or shifts it towards extracellular release. Peripheral biomarkers may assist this process, but blood- or cerebrospinal-fluid-derived measurements do not necessarily reproduce brain-derived changes (de la Monte et al., 2025; Bellini et al., 2022b). EV-based biomarkers likewise require standardised isolation and characterisation (Welsh et al., 2024). A testable translational hypothesis is that patients with predominant defects in lysosomal acidification may respond differently from those whose principal limitation lies in hydrolase activity, lipid storage, membrane integrity or axonal transport. Prospective biomarker studies will be required to determine whether stratification by degradative bottleneck provides information beyond conventional clinical diagnosis.

The final development package should connect product quality, brain and organellar exposure, lysosomal function, dynamic autophagic flux, complete substrate degradation, extracellular release, direct seeding activity and repeated-dose safety. These endpoints should be measured within linked experimental systems rather than assembled from unrelated studies. A platform can reasonably be described as restoring pathological-protein clearance only when it improves terminal degradation without increasing the extracellular pool of biologically active protein or imposing a new burden on the lysosomal system. These requirements are integrated into the hierarchical validation framework shown in Figure 4.

**Figure 4:**
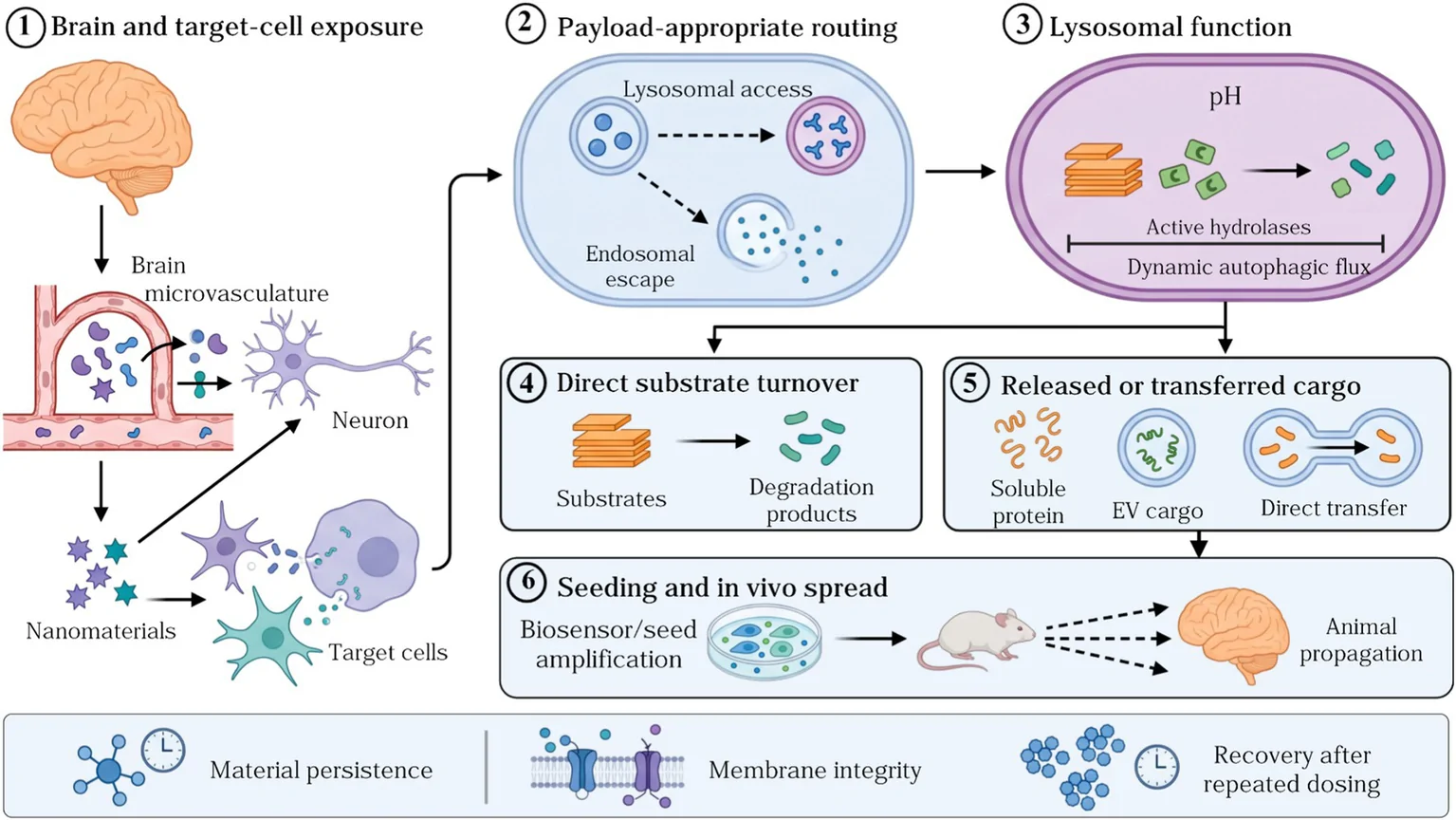
Linked evaluation of lysosome-directed nanomaterials from brain exposure to substrate fate and seeding. The proposed framework connects delivery, organelle function and pathological-protein fate through six linked assessments. (1) Establish brain and target-cell exposure, distinguishing vascular localisation from delivery to relevant neural cells. (2) Verify payload-appropriate subcellular routing: lysosomal access or endosomal escape depends on the intended site of action. (3) Assess lysosomal pH, active hydrolases and dynamic autophagic flux. Two complementary assessment arms then examine (4) direct turnover of the pathological substrate and formation of degradation products, and (5) released or transferred cargo, distinguishing soluble protein, EV-associated cargo and direct intercellular transfer. (6) Test seeding activity using biosensor, seed-amplification or recipient-cell aggregation assays, and evaluate animal propagation when claims concern *in vivo* spread. The lower strip highlights material persistence, membrane integrity and recovery after repeated dosing as requirements across the framework. Arrows indicate the organisation of validation steps and complementary measurements, rather than an established therapeutic sequence. Success in delivery or an upstream lysosomal marker does not establish complete degradation, reduced release or seed inactivation. EV, extracellular vesicle. Created with BioRender.com

## 8 Discussion and perspectives

Nanomaterial-mediated uptake does not establish pathological-protein clearance. Productive disposal requires delivery to a competent lysosomal compartment, sufficient acidification and hydrolase activity, and autophagic input matched to terminal processing capacity. Lipid handling, membrane integrity, ion balance and cellular bioenergetics determine whether this capacity can be sustained. When these functions fail, cargo may persist intracellularly or be redirected towards extracellular-vesicle release, lysosomal exocytosis or direct intercellular transfer. Reduced intracellular or total protein abundance should therefore be interpreted alongside direct turnover, extracellular cargo and seeding assays.

The current evidence supports different parts of this model with markedly different levels of confidence. The most convincing nanomaterial studies show that lysosomal acidity can be restored, hydrolase activity can recover and autophagic processing can be improved, with accompanying reductions in Aβ, tau, α-synuclein or polyglutamine-related pathology. Evidence for the recovery of dynamic autophagic flux is also reasonably strong in selected systems, although many reports still rely heavily on static LC3, p62 or pathway-signalling measurements. Changes in pathological-protein burden are widely documented, but the mechanisms underlying those changes are not always resolved. By comparison, the case for nanomaterial-mediated correction of lysosomal lipid processing and membrane stability is promising but less complete. Studies involving the GBA1–GCase axis, BMP-dependent enzyme activity, cholesterol mobilisation, lipid-droplet burden and lysosomal membrane stress indicate that these processes can shape sustained clearance. Even so, relatively few platforms have linked such changes directly to long-term pathological-protein turnover in neural models. The largest gap lies further downstream. Studies that measure lysosomal repair, complete substrate degradation, extracellular release and residual seeding activity within the same experimental system remain rare. Most nanomaterial interventions therefore support claims of reduced protein burden, restricted aggregation or improved clearance-related function. Far fewer justify stronger conclusions such as seed inactivation or interruption of brain-wide propagation.

Future studies should link delivery, organelle rescue and pathological-substrate fate within a single experimental chain. Regional and cell-specific exposure should be connected to lysosomal pH, V-ATPase activity, hydrolase maturation and dynamic autophagic flux, followed by direct measurements of protein turnover and extracellular release. Residual activity should then be tested in recipient-cell, seed-amplification or *in vivo* propagation assays. These efficacy endpoints must be evaluated alongside material persistence, ion release, membrane damage and recovery after repeated dosing. The most informative platforms will not simply maximise brain uptake or autophagy. They will match the intervention to the pathological conformer, restore lysosomal function without creating a new organelle burden and reduce both intracellular substrate and extracellular seeding competence.
